# Multi-omics analysis of polysaccharide accumulation and associated metabolic reprogramming across developmental stages of *Ganoderma tsugae*

**DOI:** 10.3389/fmicb.2026.1856773

**Published:** 2026-06-17

**Authors:** Weihan Kong, Peng Wang, Xueping Kang, Ya Yu, Lei Xia, Xiao Tan, Zhiqiang Yang, Jiawei Wen, Di Liu

**Affiliations:** 1 Agricultural College, Yanbian University, Yanji, Jilin, China; 2 Jilin Academy of Agricultural Sciences (Northeast Agricultural Research Center of China), Changchun, Jilin, China; 3 Jilin Province Shanzhiyuan Ecological Agriculture Co., Ltd., Changchun, Jilin, China

**Keywords:** fruiting bodies, *Ganoderma tsugae*, metabolic reprogramming, metabolomics, polysaccharides, transcriptomics

## Abstract

**Introduction:**

Polysaccharides are important bioactive components of *Ganoderma tsugae* fruiting bodies; however, the developmental dynamics and molecular processes associated with their accumulation remain poorly understood. This study aimed to elucidate polysaccharide-associated metabolic remodeling during fruiting body development under oak-log biomimetic cultivation.

**Methods:**

Five developmental stages were examined, including primordia (T1), immature fruiting body (T2), pre-basidiospore discharge (T3), basidiospore discharge (T4), and the end of basidiospore discharge (T5). Crude polysaccharide content was determined, and transcriptomic and untargeted metabolomic analyses were integrated to identify key pathways, genes, and metabolites associated with polysaccharide accumulation. Selected genes were further validated by qRT-PCR.

**Results:**

Crude polysaccharide content increased from T1 to T3, reached a maximum at T3 (0.90 g/100 g), and subsequently declined. A total of 5,676 differentially expressed genes and 911 differentially accumulated metabolites were identified, with clear stage-dependent separation at both transcriptomic and metabolomic levels. Integrated KEGG analysis indicated that starch and sucrose metabolism was the core pathway associated with polysaccharide accumulation, whereas galactose metabolism functioned as a supplementary module contributing to precursor supply and structural diversification. Dynamic expression of *FKS1* and multiple glycoside hydrolase-related genes, together with changes in sugar phosphates and disaccharide metabolites, suggested coordinated regulation of substrate degradation, precursor supply, glycan elongation, and carbon redistribution. The qRT-PCR results were consistent with the RNA-seq data.

**Discussion:**

These findings indicate that polysaccharide accumulation in *G. tsugae* fruiting bodies is a developmentally coordinated process rather than the result of a single isolated biosynthetic step. The T3 stage appears to represent a key transition point for polysaccharide accumulation and carbon metabolic reprogramming. This study provides a molecular and metabolic framework for understanding polysaccharide accumulation during *G. tsugae* development and offers potential candidate genes and pathways for future functional validation.

## 1 Introduction

*Ganoderma tsugae* Murrill is a rare medicinal fungus mainly distributed in the cool temperate zone of the Changbai Mountain region in China. It usually grows in mixed coniferous-broadleaf forests at altitudes of 700–1,400 m, where it colonizes stumps or decaying wood of tree species such as red pine, spruce, fir, and larch (Luo et al., 2024). As a valuable traditional medicinal material, *G. tsugae* is rich in terpenoids, polysaccharides, sterols, adenosine, and other bioactive metabolites, and exhibits antioxidant, antitumor, memory-enhancing, antitussive, and antiasthmatic activities. Its medicinal value has therefore been widely recognized (Lin and Yang(eds), 2019; Luo et al., 2024). Among these constituents, *Ganoderma* polysaccharides are considered one of the most important functional components reported in the genus and are major contributors to its diverse pharmacological properties (Yang et al., 2025).

Fungal polysaccharides are mainly composed of β-glucans and heteropolysaccharides and represent the most abundant compounds in fungal cell walls (Ruiz-Herrera and Ortiz-Castellanos, 2019). They possess highly branched architectures and strong structure-function relationships (Yang et al., 2025). With advances in glycochemistry, structural elucidation, and molecular-mechanistic studies, increasing progress has been made in understanding the structural composition of *Ganoderma* polysaccharides, their recognition by immune receptors, and downstream signaling pathways. Current studies mainly focus on improved extraction strategies and structural characterization. Reported extraction methods for *Ganoderma lucidum* polysaccharides include hot-water extraction (Khalil and Lukasiewicz, 2024), ultrasound-assisted extraction (Cui et al., 2018), enzyme-assisted extraction (Zhu et al., 2014), microwave-assisted extraction (Smiderle et al., 2017), supercritical-fluid-assisted extraction (Zhao et al., 2021), ultrasound-microwave synergistic extraction (Huang and Ning, 2010), and microbial fermentation (Wan Mohtar et al., 2016). For structural elucidation, methylation analysis coupled with GC-MS (Pan et al., 2012; Liu et al., 2024) and one-dimensional/two-dimensional nuclear magnetic resonance (1D/2D NMR) spectroscopy (Luo et al., 2024) are widely used, with methylation analysis and NMR serving as core techniques for identifying glycosidic linkages, anomeric configurations, and main/side-chain relationships. Monosaccharide composition and functional-group features are commonly analyzed after acid hydrolysis by HPLC, HPAEC-PAD, or GC/GC-MS (Zhang et al., 2018), together with FT-IR to characterize hydroxyl, carboxyl, sulfate, and glycosidic bond features, and GPC, SEC, or HPGPC to determine molecular weight and chain conformation (Luo et al., 2024).

Previous studies have shown that the biological activities of *Ganoderma* polysaccharides are closely associated with their structural characteristics, including monosaccharide composition, molecular weight, glycosidic linkage, branching degree and chain conformation (Zhang et al., 2023). Among them, β-D-glucans are considered one of the representative bioactive polysaccharide groups in *Ganoderma* species. These polysaccharides commonly contain β-(1→3)-D-glucan backbones with β-(1→6)-linked side chains and have been associated with immunomodulatory, antitumor, antioxidant and anti-inflammatory activities (Zhang et al., 2023). In addition to β-D-glucans, a fucose-containing glycoprotein fraction isolated from *G. lucidum* water-soluble extracts has been reported to stimulate spleen cell proliferation and cytokine expression, suggesting that heteropolysaccharide- or glycoprotein-type fractions may also contribute to the immunological activity of *Ganoderma* polysaccharides (Wang et al., 2002). Moreover, fucose-containing polysaccharides from Reishi mushroom have been shown to induce antibodies against tumor-associated Globo H-series epitopes, further supporting the functional relevance of structurally specific polysaccharide fractions (Liao et al., 2013). Therefore, developmental-stage-dependent changes in polysaccharide accumulation may have potential implications for the quality evaluation and functional utilization of *Ganoderma* fruiting bodies.

*Ganoderma* polysaccharide biosynthesis is increasingly recognized as a coordinated process involving carbon source conversion, nucleotide-sugar precursor supply, glycan assembly, cell wall remodeling, and extracellular export (Li et al., 2015; Zhang et al., 2023). Following entry into central carbon metabolism, carbon substrates are converted into activated sugar donors such as UDP-Glc via key enzymes including phosphoglucomutase (PGM) and UDP-glucose pyrophosphorylase (UGP), while other nucleotide sugars, such as GDP-Man, UDP-Gal, and UDP-GlcA, also contribute to structural heterogeneity (Li et al., 2015; Wang et al., 2023). Glycosyltransferases, represented by 1,3-β-glucan synthase (GLS), drive backbone extension and branching, whereas glycoside hydrolases and GPI-anchored cell wall proteins participate in polysaccharide remodeling and release (Chen et al., 2024). However, an integrated model linking precursor allocation, glycan assembly, cell wall dynamics, and polysaccharide export is still lacking. Clarifying this regulatory network will be essential for understanding structure formation and supporting strain improvement, fermentation control, and industrial production.

In recent years, high-throughput omics approaches, especially the integration of transcriptomics (RNA-seq) and metabolomics (LC-MS/MS), have become powerful tools for deciphering complex developmental processes and the accumulation of bioactive metabolites (Pinu et al., 2019; Zhang et al., 2022; Yang et al., 2024). Recent evidence indicates that fungal polysaccharide biosynthesis is not a single enzymatic event; rather, it involves coordinated regulation of central carbon metabolism, nucleotide-sugar precursor supply, glycosyl transfer, glycoside hydrolysis, and cell-wall assembly (Guo et al., 2025). Within this framework, *G. lucidum* has gradually become an important model for studying polysaccharide biosynthesis and precision regulation in edible and medicinal fungi. In the genus *Ganoderma*, research has shifted from descriptive comparisons toward mechanistic dissection (Wang et al., 2023), through integrated transcriptomic and proteomic analyses, found that several glycoside hydrolase families associated with cell-wall degradation were significantly upregulated under high-polysaccharide-yield conditions, suggesting that cell-wall polysaccharide turnover and polysaccharide release are closely linked to yield formation (Chen et al., 2024) further demonstrated that α-1,3-glucosyltransferase can markedly alter *Ganoderma* polysaccharide yield by affecting genes involved in UDP-glucose synthesis, glycosyltransferases, and cell-wall structure. Meanwhile, (Meng et al., 2022) showed that integrated transcriptome-metabolome analysis across developmental stages can effectively identify key transcription factors and metabolic pathways associated with active-component accumulation, highlighting the value of multi-omics integration for elucidating coordinated developmental and metabolic regulation in *Ganoderma*.

In the present study, oak logs, which are commonly used in production, were employed as the cultivation substrate, and five key developmental stages of *G. tsugae* fruiting bodies—primordia, immature fruiting body, pre-basidiospore discharge, basidiospore discharge and end of basidiospore discharge—were selected for investigation. By quantifying the dynamic changes in polysaccharide content and integrating transcriptomic sequencing with untargeted metabolomics, we established a combined metabolome-transcriptome framework to reveal the molecular basis of polysaccharide biosynthesis and metabolic remodeling during *G. tsugae* development, thereby providing a theoretical basis for resource exploitation and quality-oriented cultivation control of *G. tsugae*.

## 2 Materials and methods

### 2.1 Materials

The *G. tsugae* strain used in this study was JNJTG43, provided by the Institute of Economic Plants, Jilin Academy of Agricultural Sciences (Northeast Innovation Center of Agricultural Science and Technology, China). This cultivated strain was domesticated from a wild isolate and is preserved at the Institute of Microbiology, Chinese Academy of Sciences under strain number CGMCC No. 41686. Spawn was prepared by stepwise expansion. Mycelia were first grown on potato dextrose agar (PDA) at 25°C in the dark until the plates were fully colonized, yielding first-level spawn.

A sawdust substrate containing 78% wood chips, 20% wheat bran, 1% lime, and 1% gypsum was adjusted to approximately 60% moisture, packed into polypropylene bags, and sterilized at 121°C for 1 h to produce sawdust culture bags. After cooling to room temperature, first-level spawn was aseptically inoculated into the sawdust bags to produce second-level spawn. The inoculated bags were then incubated at about 25°C in darkness until the mycelia had fully colonized the substrate.

Oak logs with diameters of 18–20 cm were cut into segments 20–25 cm in length and lightly air-dried for 15–20 d in a clean, well-ventilated area. Every ten short logs were bundled together and placed in polypropylene bags, which were sterilized at 100°C under atmospheric pressure for 9 h to prepare log culture bags. After cooling, second-level spawn was aseptically inoculated into the log bags at 300 g per bag to obtain third-level spawn (Xia et al., 2024). These bags were incubated in darkness until the logs were fully colonized. The colonized log bags were then transferred to the cultivation base of Changbaishan Zhiyuan Ecological Agriculture Co., Ltd. in Changbai County, Jilin Province, China. The polypropylene bags were removed, half of each log was buried in the soil of a Korean pine forest, and a small amount of pine needles was used as cover, thereby establishing an under-forest biomimetic cultivation system to induce fruiting body formation.

**FIGURE 1:**
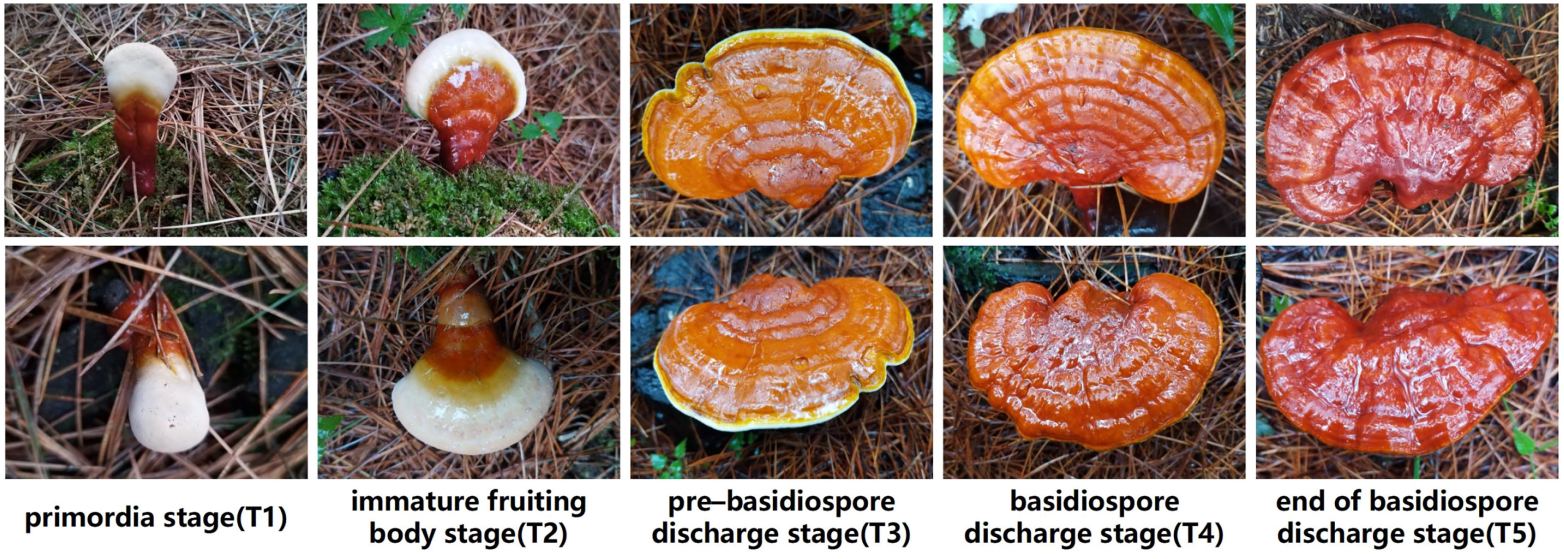
Morphological characteristics of *G. tsugae* fruiting bodies at five developmental stages. T1, primordia; T2, immature fruiting body; T3, pre-basidiospore discharge; T4, basidiospore discharge; T5, end of basidiospore discharge.

At five developmental stages: primordia (T1), immature fruiting body (T2), pre-basidiospore discharge (T3), basidiospore discharge (T4) and end of basidiospore discharge (T5), six independently grown fruiting bodies were collected as biological replicates (Figure 1). Each fruiting body was sampled individually. One portion was used for crude polysaccharide determination, and the other was immediately frozen in liquid nitrogen for LC-MS/MS analysis and RNA extraction, thereby ensuring the reliability and reproducibility of the experiments. Three independent biological replicates per stage were used for crude polysaccharide determination and transcriptome sequencing, whereas six independent biological replicates per stage were used for metabolomic analysis.

### 2.2 Extraction and determination of crude polysaccharides

Fresh *G. tsugae* samples were rinsed immediately with distilled water to remove surface impurities, blotted dry, and rapidly frozen in liquid nitrogen. The frozen material was crushed and ground into a fine powder, pretreated with absolute ethanol, refluxed to remove low-molecular-weight interfering substances, and dried at 40°C to obtain a pretreated powder. Crude polysaccharides were determined by the phenol-sulfuric acid method using glucose as the standard. Different volumes of glucose working solution (0–1.0 mL) were adjusted to 1.0 mL with distilled water, followed by sequential addition of 1.0 mL phenol solution and 5.0 mL concentrated sulfuric acid. After standing for 10 min, the mixture was vortexed, reacted in a 30°C water bath for 20 min, and measured at 490 nm to establish the standard curve.

Samples were first pretreated with ethanol to remove low-molecular-weight interferents. After wetting with water, absolute ethanol was added, the sample was ultrasonically extracted for 30 min, and centrifuged at 4,000 r/min for 10 min using an Eppendorf Centrifuge 5810 R centrifuge (Eppendorf AG, Hamburg, Germany); the supernatant was discarded. The residue was washed with 80% ethanol and then subjected to aqueous extraction. Water was used as the solvent, and ultrasonic extraction was performed at 120 W for 30 min and repeated twice. The filtrates were combined and brought to a fixed volume. An aliquot of the extract was diluted to 1.0 mL with distilled water, mixed with 1.0 mL phenol solution and 5.0 mL concentrated sulfuric acid, allowed to stand for 10 min, vortexed, reacted at 30°C for 20 min, and measured at 490 nm (Gao et al., 2022). Polysaccharide content was calculated from the glucose standard curve and expressed as mass fraction W (g/100 g) according to Equation (1):


$$W=\frac{m_{1}\times V_{1}}{m_{2}\times V_{2}}\times 0.9\times 10^{-4}$$ (1)

where m_1_ is the sugar content of the assay solution determined from the standard curve (μg), V_1_ is the final volume of the sample extract (mL), V_2_ is the aliquot volume used for color development (mL), m_2_ is the sample mass (g), and 0.9 is the correction factor converting glucose to glucan.

Crude polysaccharide contents are presented as mean ± standard deviation. Differences among developmental stages were first assessed by one-way ANOVA. When the assumptions of ANOVA were satisfied, Duncan’s multiple range test was further used for pairwise comparisons, and *P* < 0.05 was considered statistically significant.

### 2.3 RNA extraction, library construction, and sequencing

Total RNA was extracted from liquid-nitrogen-ground *G. tsugae* fruiting body samples using the Omega Plant RNA Kit (Omega Bio-Tek, R6827) according to the manufacturer’s protocol. Briefly, approximately 100 mg of frozen tissue powder was lysed with Buffer RCL containing β-mercaptoethanol, followed by genomic DNA removal using a gDNA Filter Column and RNA purification with a HiBind RNA Mini column. RNA concentration, purity, and integrity were assessed by agarose gel electrophoresis, NanoDrop spectrophotometry, Qubit 2.0 Fluorometer, and Agilent 2100 Bioanalyzer (You et al., 2021).

For library construction, poly(A)+ mRNA was enriched using mRNA Capture Beads, fragmented, and then used for first- and second-strand cDNA synthesis. Sequencing libraries were constructed using the Hieff NGS^®^ Ultima Dual-mode mRNA Library Prep Kit through end repair, A-tailing, adapter ligation, PCR amplification, and purification. Library quality was evaluated before sequencing, and sequencing was performed by Gene Denovo Biotechnology Co., Ltd. (Guangzhou, China).

Raw sequencing data were processed with fastp v0.18.0^1^ to remove adapter contamination, reads with high N content, polyA reads, and low-quality reads. The resulting clean reads were then aligned to an rRNA database using bowtie2 v2.28^2^ to remove residual rRNA reads. The remaining reads were mapped to the reference genome using HISAT2 v2.1.0.^3^ Based on the alignment results, transcripts were reconstructed with StringTie v1.3.4,^4^ and gene expression levels were quantified by RSEM v1.2.19.^5^ Expression abundance was reported as both raw read counts and TPM.

Differential expression analysis was performed using raw read counts as input with DESeq2.^6^ Group comparisons were carried out with DESeq2, and genes with |log_2_ FC| ≥ 1 and FDR ≤ 0.05 were defined as differentially expressed genes (DEGs). Because this study focused on differences among developmental stages, the groupwise comparison results were used as the basis for DEGs analysis in the main text. TPM values were used only for expression display and heatmap visualization and not as input for differential analysis.

### 2.4 Metabolite extraction and analysis

Approximately 100 mg of liquid-nitrogen-ground *G. tsugae* fruiting body tissue was mixed with 500 μL of precooled 80% methanol in water, vortexed thoroughly, incubated on ice for 5 min, and centrifuged at 15,000 g and 4°C for 20 min. An appropriate volume of the supernatant was diluted with LC-MS-grade water to a final methanol concentration of 53%, centrifuged again at 15,000 g and 4°C for 20 min, and the resulting supernatant was collected for LC-MS/MS analysis (Sellick et al., 2011; Yuan et al., 2012). QC samples were prepared by pooling equal volumes of all biological samples to evaluate instrument stability. Blank samples consisted of 53% methanol in water processed in parallel with the same pretreatment procedure.

UHPLC-MS/MS analysis was performed on a Vanquish UHPLC system coupled to an Orbitrap Q Exactive HF-X mass spectrometer (Thermo Fisher Scientific, Germany). Separation was achieved on a Hypersil Gold column (100 × 2.1 mm, 1.9 μm) at 40°C with a flow rate of 0.2 mL/min. In positive-ion mode, mobile phase A was 0.1% formic acid in water and mobile phase B was methanol. In negative-ion mode, mobile phase A was 5 mM ammonium acetate (pH 9.0) and mobile phase B was methanol. The gradient program was 0–1.5 min, 2% B; 1.5–12.0 min, 2–100% B; 12.0–14.0 min, 100% B; and 14.1–17.0 min, return to 2% B. The mass scan range was set to m/z 100–1,500. The spray voltage was 3.5 kV, sheath gas flow was 35 psi, auxiliary gas flow was 10 L/min, ion transfer tube temperature was 320°C, S-lens RF level was 60, and auxiliary gas heater temperature was 350°C. Data-dependent MS/MS acquisition was performed in both positive- and negative-ion modes.

Raw mass spectrometry files (.raw) were converted to mzXML format with ProteoWizard (Chambers et al., 2012), followed by peak extraction, alignment, and quantification using XCMS (Smith et al., 2006). Metabolite annotation was based on retention time, mass-to-charge ratio, adduct information, and high-quality MS/MS database matching within a mass tolerance of 10 ppm. After removal of background ions using blank samples, the raw peak areas were normalized to obtain relative peak areas for subsequent statistical analyses. Using FDR ≤ 0.05, |log_2_FC| ≥ 1 and VIP ≥ 1 as the screening criteria for differentially accumulated metabolites (DAMs). Chemical classification of metabolites was performed using ClassyFire.^7^

### 2.5 Integrative transcriptome-metabolome analysis

The O2PLS model was generated in R software (R Foundation for Statistical Computing, Vienna, Austria)^8^ using the OmicsPLS package to integrate transcriptomic and metabolomic data (el Bouhaddani et al., 2016). For integrative transcriptome–metabolome analysis, the O2PLS model was constructed using the same three biological replicates per developmental stage for which both RNA-seq and metabolomic data were available (*n* = 15 paired samples in total). The additional metabolomic replicates were used for metabolite profiling, differential metabolite screening, and quality assessment, but were not included in the paired O2PLS model. The optimal number of components was determined by cross-validation, and the best model was used for integrative analysis. Spearman correlation coefficients were calculated, and gene-metabolite pairs were ranked in descending order according to the absolute correlation coefficient. The top 250 gene-metabolite pairs with absolute Spearman correlation coefficients > 0.5 were used to construct the metabolite-transcript network with the igraph package in R. PCA plots, volcano plots, heatmaps, UpSet plots, bar plots, and network plots were generated in R using packages including ggplot2, pheatmap, UpSetR, ggpubr, OmicsPLS, and igraph.

### 2.6 qRT-PCR validation

Based on the transcriptomic and metabolomic analyses, 10 representative candidate DEGs were selected for qRT-PCR validation (Liu et al., 2020). Gene-specific primer pairs were designed using the NCBI database. Reverse transcription and quantitative PCR were conducted using Hifair AdvanceFast One-step RT-gDNA Digestion SuperMix for quantitative reverse transcription (qPCR) and Hifair qPCR SYBR Green Master Mix (High Rox Plus) (Yeasen Biotechnology, Shanghai, China) according to the manufacturer’s instructions. qRT-PCR was carried out on a StepOne Plus instrument under the following program: 95°C for 5 min; 40 cycles of 94°C for 15 s, 58°C for 30 s, and 70°C for 30 s. Three technical replicates were set for each sample. The pre-experiment has verified that 18S gene is selected as the internal reference gene for expression normalization (Xiang et al., 2018), and relative expression levels were calculated using the 2^–ΔΔCt^ method.

## 3 Results

### 3.1 Dynamic changes in crude polysaccharide content

**FIGURE 2:**
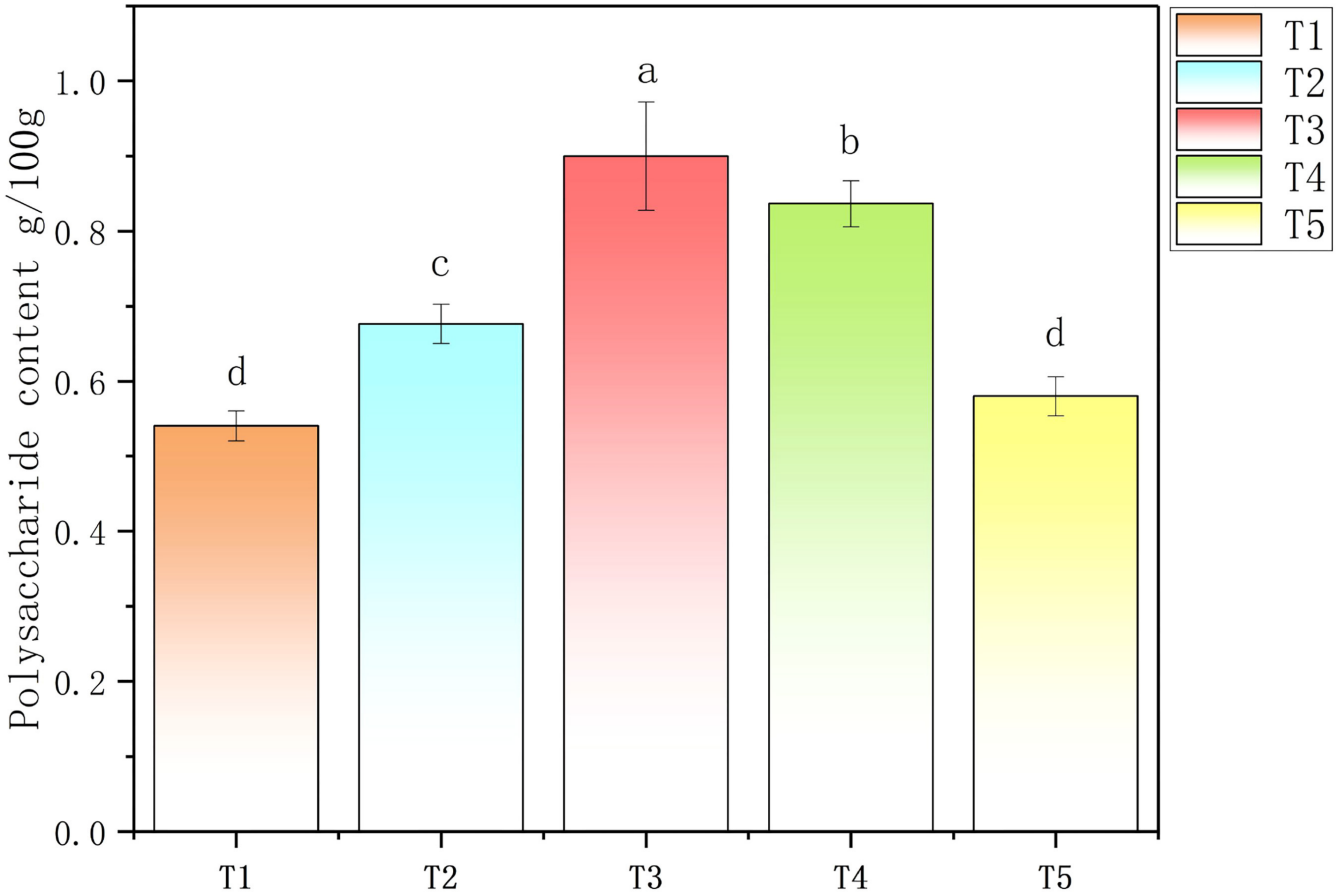
Crude polysaccharide content of *G. tsugae* fruiting bodies across developmental stages. Values are mean ± SD. Different lowercase letters indicate significant differences among stages at *P* < 0.05. T1–T5 are defined as in Figure 1.

As shown in Figure 2, polysaccharide content exhibited an overall trend of first increasing and then decreasing during development. It rose gradually from T1 to T3, reached a maximum at T3, and then declined slightly at T4 and T5. Specifically, T3 showed the highest polysaccharide content (0.90 g/100 g), significantly higher than that at all other stages. T4 ranked second at 0.84 g/100 g, followed by T2 at 0.68 g/100 g, whereas T1 and T5 showed the lowest levels, at 0.54 and 0.58 g/100 g, respectively, with no significant difference between them. Statistical analysis indicated that the polysaccharide content at T3 was significantly higher than those at T1 and T5 (*P* < 0.01), being 1.64- and 1.55-fold greater, respectively. The T4 level was also significantly higher than those at T2 and T5 (*P* < 0.05), indicating that polysaccharide accumulation was more active during the middle developmental stages.

### 3.2 Quality evaluation of transcriptomic and metabolomic data

#### 3.2.1 Quality evaluation of transcriptomic data

**FIGURE 3:**
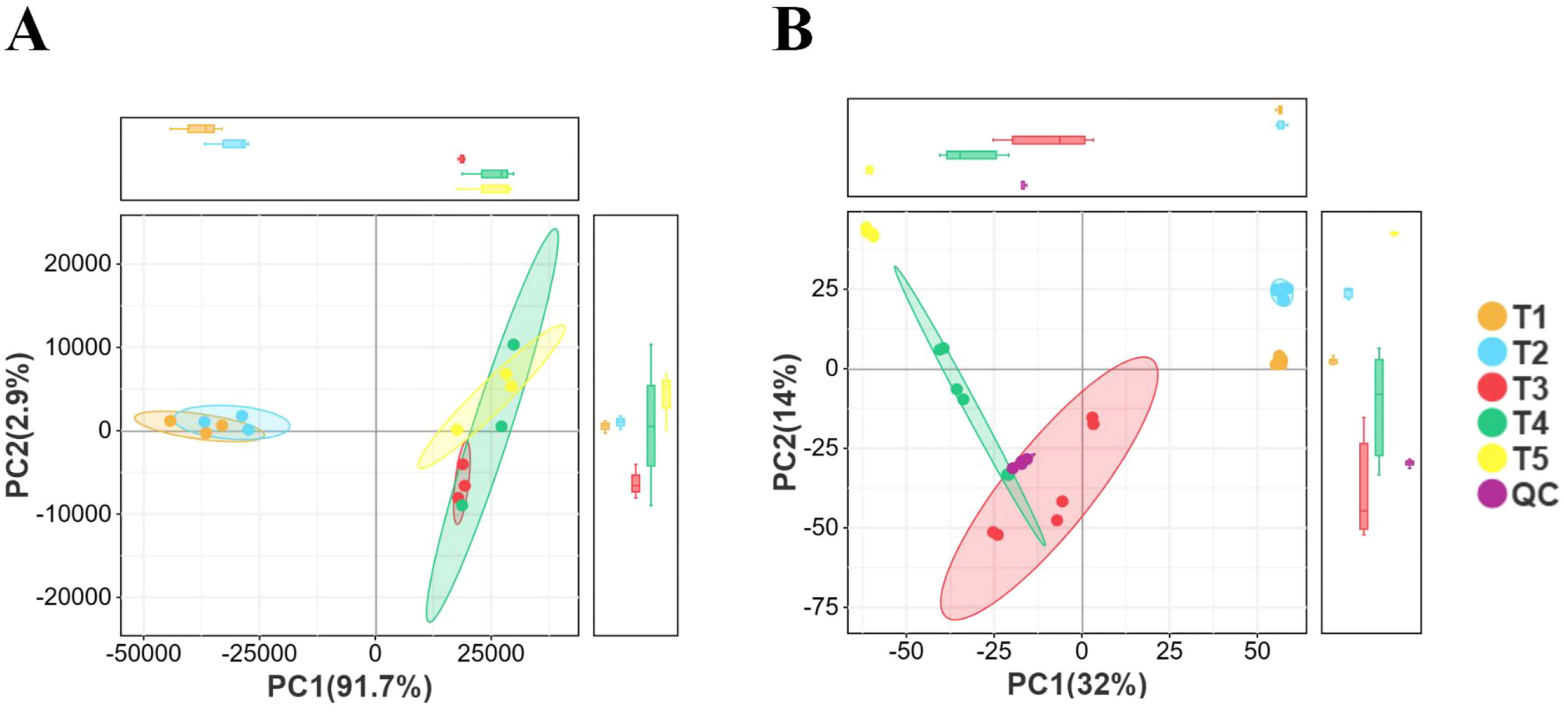
PCA of transcriptomic and metabolomic profiles of *G. tsugae* across developmental stages. **(A)** Transcriptomic PCA based on 15 RNA-seq samples. **(B)** Metabolomic PCA based on 30 LC-MS/MS samples and QC samples. PC1 and PC2 indicate the first two principal components. QC, quality control.

Principal component analysis (PCA) groups samples with similar gene-expression profiles; shorter intersample distances indicate higher similarity. PCA of the 15 transcriptome samples (Figure 3A) showed that the samples were separated into three clusters. T1 and T2 were relatively close to each other and could be approximately grouped together, whereas these two groups were clearly distant from T3, T4 and T5, indicating marked transcriptional differences between early and middle/late developmental stages. T3 and T5 were relatively close in PCA space, especially along PC1, although some separation remained along PC2, suggesting more similar but not identical expression patterns. For T1, T2, T3 and T5, the three biological replicates of each stage clustered tightly, indicating good within-group stability and repeatability. In contrast, the three T4 samples were more dispersed, which may reflect variation in the onset of basidiospore discharge among samples, causing some to resemble either T3 or T5 fruiting bodies more closely.

#### 3.2.2 Quality evaluation of metabolomic data

The metabolomic PCA results (Figure 3B) showed that the first two principal components, PC1 and PC2, explained 32 and 14% of the total variance, respectively, accounting for 46% of the overall variation. Based on these two principal components, 30 metabolomic samples from the five developmental stages and six QC samples were clearly separated into six distinct groups (Figure 3B). The QC samples clustered tightly, indicating good analytical stability and reliability. Only limited overlap was observed between T3 and T4, suggesting that intergroup metabolic differences were still discernible even under an unsupervised model.

To further resolve interstage differences, orthogonal partial least squares-discriminant analysis (OPLS-DA) was performed. The results showed good clustering within groups and no overlap among groups, indicating marked metabolic differences between developmental stages (Supplementary Figure 1). The high R2X, R2Y, and Q2 values indicated strong model fit and predictive ability. To assess possible overfitting, 200 permutation tests were conducted. In all six group comparisons, R2Y and Q2 were > 0.9, and the Q2 intercepts from permutation testing were all below zero (Supplementary Figure 2), confirming that the models were not overfitted and were stable and reliable.

### 3.3 Analysis of DEGs and DAMs across developmental stages

#### 3.3.1 Screening and distribution patterns of DEGs

**FIGURE 4:**
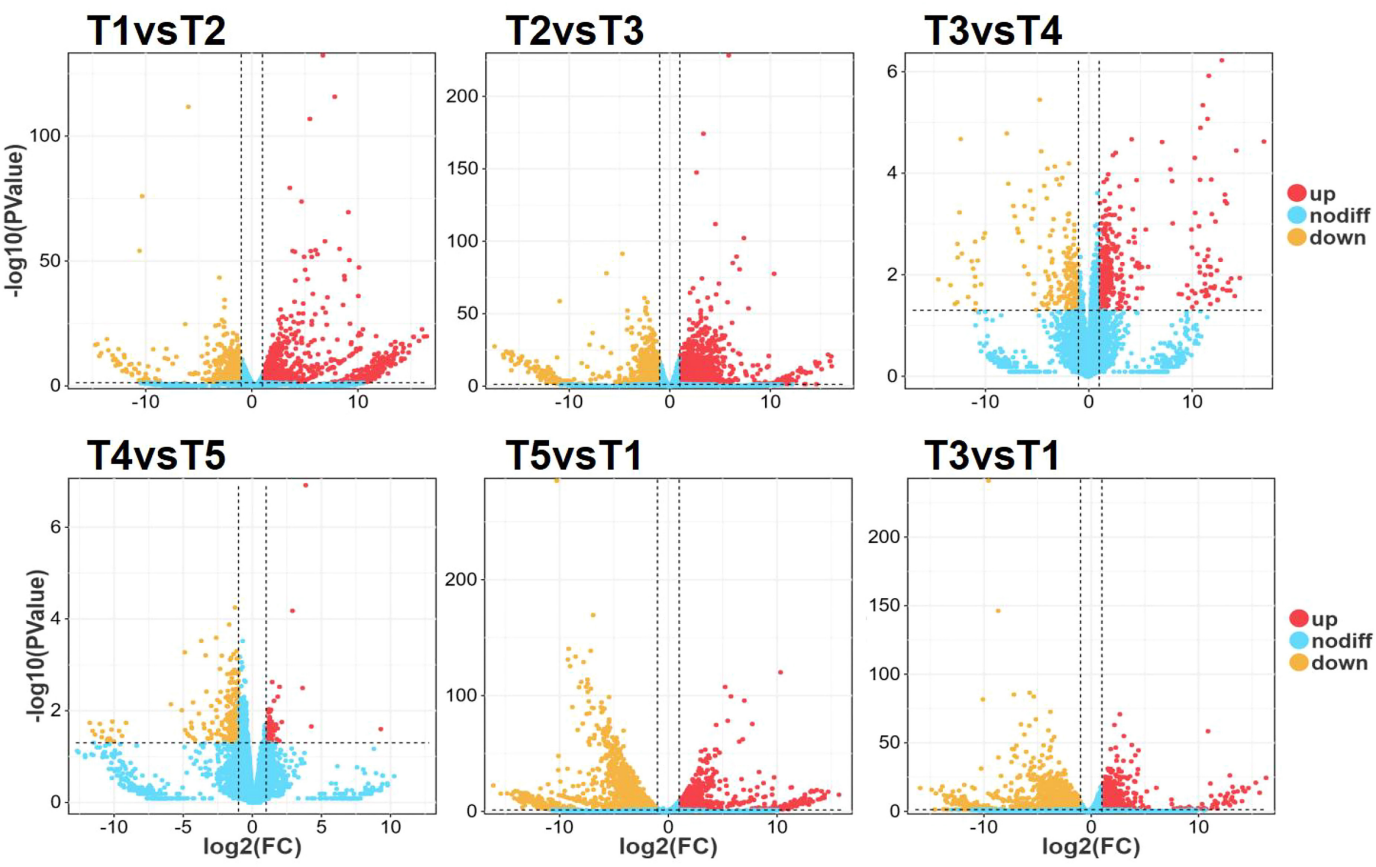
Volcano plots of DEGs among developmental stages of *G. tsugae*. Each point represents one gene. Red and blue points indicate significantly upregulated and downregulated genes, respectively. DEGs were identified using |log2 FC| ≥ 1 and FDR ≤ 0.05. FC, fold change; FDR, false discovery rate.

Pairwise differential analyses were first performed between adjacent developmental stages (T1 vs. T2, T2 vs. T3, T3 vs. T4, and T4 vs. T5), and were supplemented by comparisons between the terminal and initial stages (T5 vs. T1) and between the stages with the highest and lowest polysaccharide contents (T3 vs. T1) (Figure 4). In total, 5,676 DEGs were identified (Supplementary Table 1). Specifically, 1,779 DEGs were detected in T1 vs. T2 (1,010 upregulated and 769 downregulated), whereas 3,015 DEGs were identified in T2 vs. T3 (2,167 upregulated and 848 downregulated), suggesting substantial transcriptional reprogramming during the transition from T2 to T3. By contrast, only 588 DEGs were identified in T3 vs. T4 (365 upregulated and 223 downregulated), and 339 DEGs were detected in T4 vs. T5 (135 upregulated and 204 downregulated), indicating a lower amplitude of transcriptional change around the basidiospore discharge period. Among the cross-stage comparisons, T5 vs. T1 produced the largest number of DEGs (3,675; 1,052 upregulated and 2,623 downregulated), demonstrating marked transcriptional divergence between the terminal and early stages. In addition, 2,634 DEGs were detected in T3 vs. T1 (695 upregulated and 1,939 downregulated), providing an important basis for identifying polysaccharide-related pathways and candidate genes.

**FIGURE 5:**
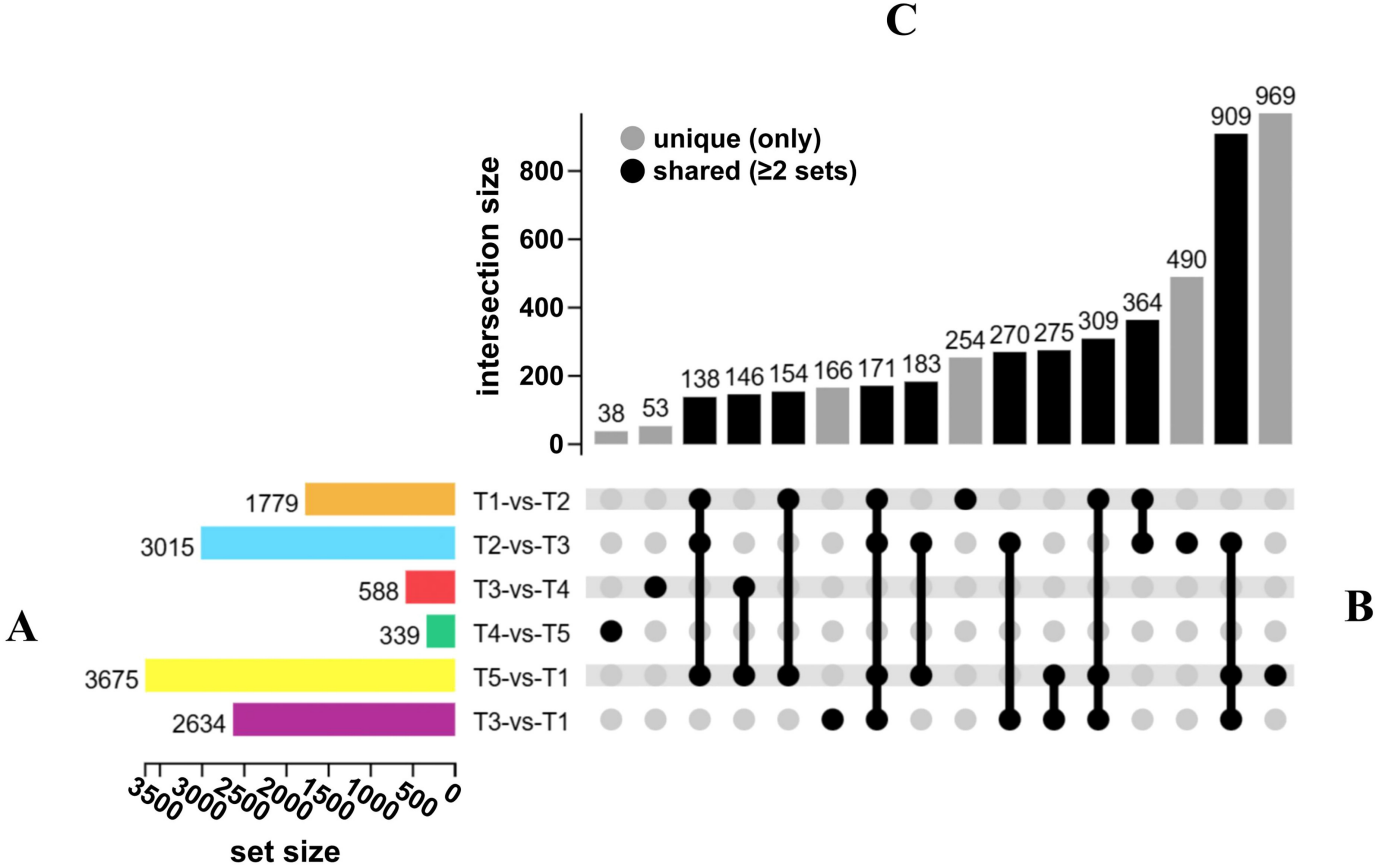
UpSet analysis of shared and stage-specific DEGs among pairwise comparisons. **(A)** Set size: horizontal bars indicate the total number of DEGs detected in each pairwise comparison. **(B)** Intersection matrix: black dots and connecting lines indicate the comparisons included in each DEG intersection; single dots indicate comparison-specific DEGs, whereas connected dots indicate DEGs shared by two or more comparisons. **(C)** Intersection size: vertical bars indicate the number of DEGs in each intersection. Gray bars denote unique DEG sets, and black bars denote shared DEG sets.

The UpSet analysis (Figure 5) showed that the numbers of DEGs in the six comparisons were 1,779, 3,015, 588, 339, 3,675, and 2,634, respectively, with T5 vs. T1, T3 vs. T1, and T2 vs. T3 exhibiting the largest transcriptional divergence. DEGs displayed both strong comparison-specificity (unique, only) and substantial overlap across comparisons (shared, ≥ 2 sets). Among the 16 displayed combinations, some DEGs were detected only in a single comparison, forming relatively large comparison-specific sets, with the largest unique set comprising 969 genes. This indicates stage-specific transcriptional responses during developmental transitions. At the same time, several comparisons shared large DEG sets, the largest shared intersection containing 909 genes; additional medium-sized shared sets of 490, 364, and 309 genes were also observed. These results suggest that, besides stage-specific regulation, a core group of DEGs is repeatedly involved in multiple developmental transitions.

#### 3.3.2 Screening and variation patterns of DAMs

**FIGURE 6:**
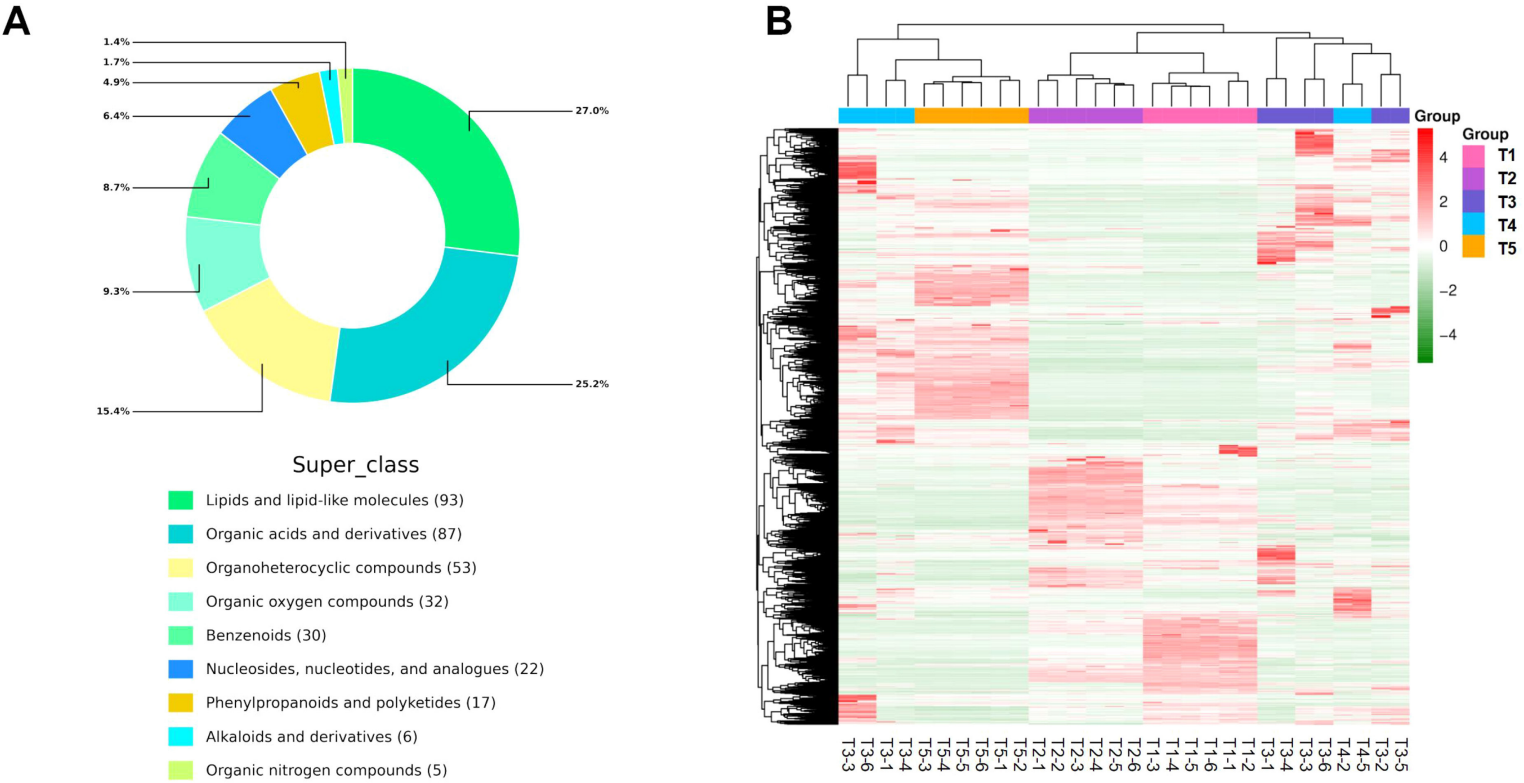
Classification and clustering of DAMs during *G. tsugae* development. **(A)** Chemical classification of annotated DAMs. **(B)** Hierarchical clustering heatmap of DAMs. Red indicates higher accumulation, and green indicates lower accumulation.

Based on untargeted LC-MS/MS metabolomics, a total of 911 DAMs were screened (Supplementary Table 2). According to chemical structural information annotated by ClassyFire,^9^ a total of 345 DAMs were assigned to nine compound categories (Figure 6A), including 93 lipids and lipid-like molecules (27.0%), 87 organic acids and derivatives (25.2%), 53 organoheterocyclic compounds (15.4%), 32 organic oxygen compounds (9.3%), 30 benzenoids (8.7%), 22 nucleosides, nucleotides, and analogs (6.4%), 17 phenylpropanoids and polyketides (4.9%), 6 alkaloids and derivatives (1.7%), and 5 organic nitrogen compounds (1.4%).

Hierarchical clustering was used to visualize the accumulation patterns of DAMs across samples. As shown in Figure 6B, samples from T1 and T2 were metabolically similar and could be clustered together, whereas samples from T3 and T4 formed another cluster. Samples from T5 differed markedly from the above two groups and were largely separated as an independent cluster. DAM levels varied substantially among developmental stages. Some DAMs accumulated at relatively high levels during the early developmental stages and decreased markedly during T4 and T5, whereas others were enriched during T3, T4 and T5 but remained low in the early stages. The accumulation patterns were highly consistent among biological replicates within the same stage, and the differences among developmental stages were much greater than those among replicates. These results indicate pronounced metabolic remodeling during fruiting body development, and confirm that developmental stage is the major driver of metabolic differentiation.

**FIGURE 7:**
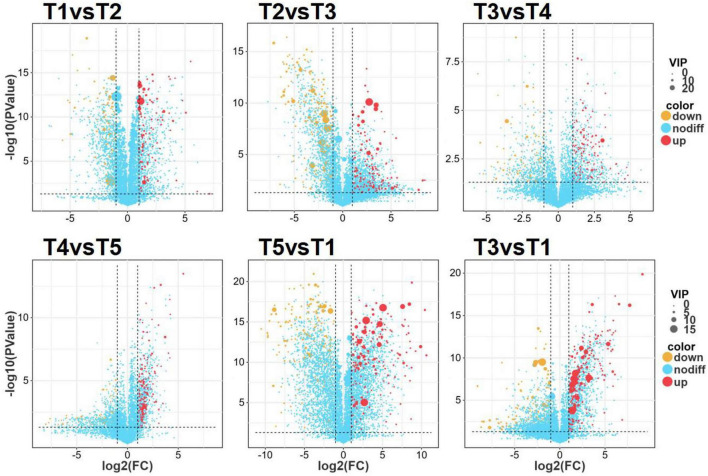
Volcano plots of differentially accumulated metabolites among developmental stages of *G. tsugae*. Each point represents one metabolite. Red and blue points indicate significantly increased and decreased metabolites, respectively. DAMs were identified using |log2 FC| ≥ 1, FDR ≤ 0.05, and VIP ≥ 1. VIP, variable importance in projection.

Volcano plots intuitively illustrated the distributions of upregulated and downregulated metabolites between developmental stages and reflected the dynamic changes in metabolic activity (Figure 7). A total of 185 DAMs were detected in T1 vs. T2 (98 increased and 87 decreased). T2 vs. T3 yielded 331 DAMs (126 increased and 205 decreased). T3 vs. T4 contained 201 DAMs (113 increased and 88 decreased). T4 vs. T5 showed 252 DAMs (169 increased and 83 decreased). T5 vs. T1 contained 369 DAMs (177 increased and 192 decreased), and T3 vs. T1 contained 338 DAMs (205 increased and 133 decreased). In T1 vs. T2, the numbers of upregulated and downregulated metabolites were similar, indicating a dynamic balance between metabolite synthesis and consumption during the transition from T1 to T2. In T2 vs. T3, downregulated metabolites outnumbered upregulated ones, suggesting an overall decline in many metabolites during the transition to T3, possibly reflecting a shift of materials and energy toward spore-related development. In T3 vs. T4 and T4 vs. T5, upregulated metabolites outnumbered downregulated metabolites, indicating relatively active metabolism during these stages, likely associated with structural remodeling of the fruiting body during and after spore release.

### 3.4 Analysis of DEGs and DAMs related to polysaccharide biosynthesis

#### 3.4.1 Expression and accumulation patterns of polysaccharide-related pathways

**FIGURE 8:**
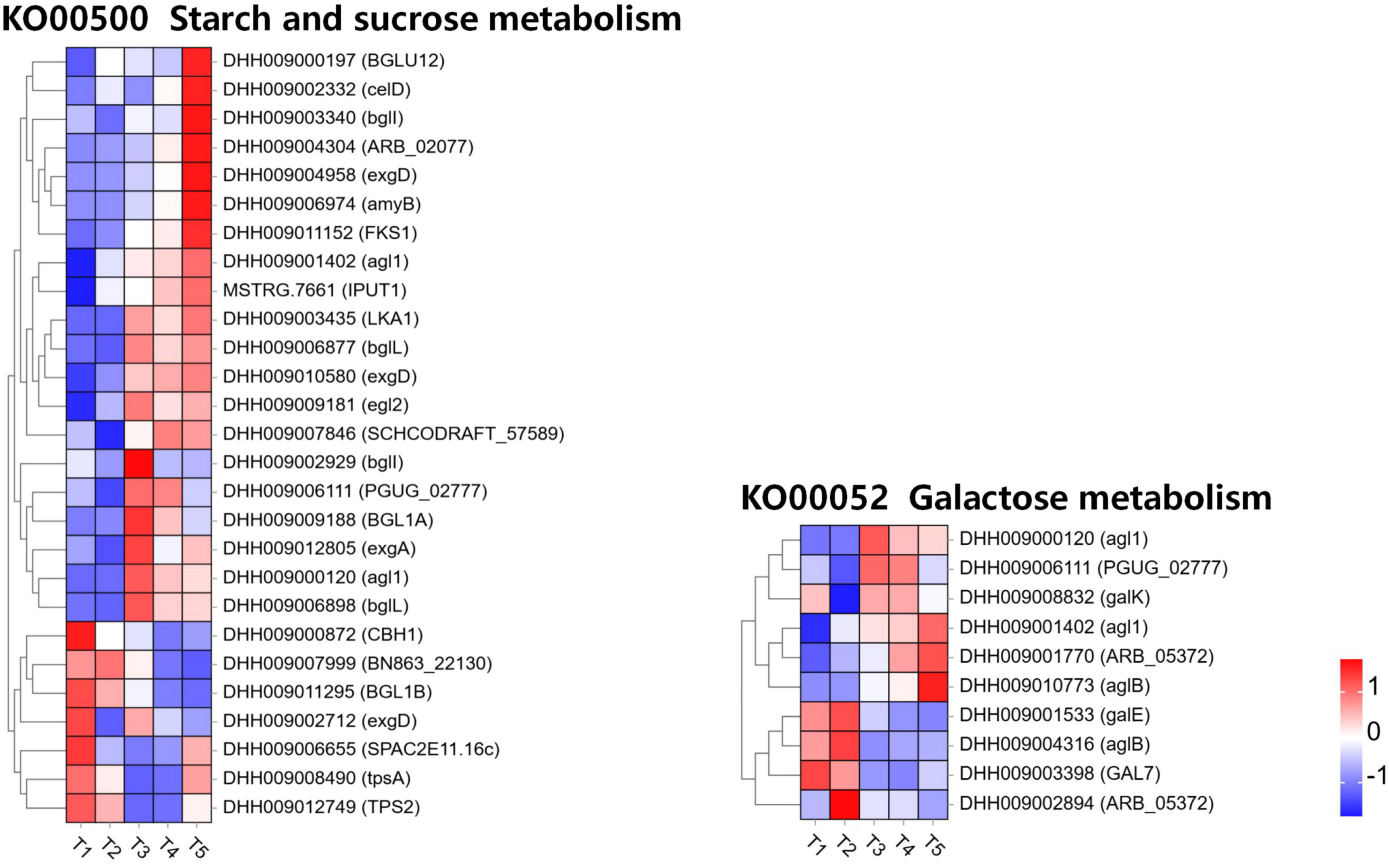
Expression patterns of DEGs involved in starch and sucrose metabolism and galactose metabolism. Heatmaps show DEGs mapped to ko00500 and ko00052. Red indicates higher expression, and blue indicates lower expression.

Because this study focused on the biosynthesis and degradation of structural units of *G. tsugae* polysaccharides, we examined carbohydrate metabolic pathways in the KEGG database (Kyoto Encyclopedia of Genes and Genomes)^10^ and found that two pathways were directly related to polysaccharide biosynthesis in *Ganoderma*: starch and sucrose metabolism (ko00500) and galactose metabolism (ko00052). Enrichment of DEGs in these pathways is shown in the gene-expression heatmap in Figure 8; detailed information is provided in Supplementary Tables 3A,B. Overall, most genes showed higher expression at later stages than at earlier stages, indicating that many genes related to polysaccharide synthesis tended to increase in expression as development progressed. These DEGs included key genes involved in cellulose, starch, sucrose, and galactose metabolism. *FKS1* encodes a β-1,3-glucan synthase-related protein involved in β-glucan chain elongation. Multiple extracellular glucanase genes, including *CBH1*, *BGL1A*, and *exgD*, play important roles in glucan hydrolysis. The trehalose biosynthesis-related genes tpsA and TPS2 may be critical for trehalose metabolism. In galactose metabolism, key genes such as galK, *ARB_05372*, and galE regulate galactose utilization through phosphorylation, transport, and downstream metabolism. The coordinated behavior of these genes indicates that *G. tsugae* possesses a diverse enzymatic system for sugar metabolism and can efficiently process and utilize multiple types of carbon sources.

**FIGURE 9:**
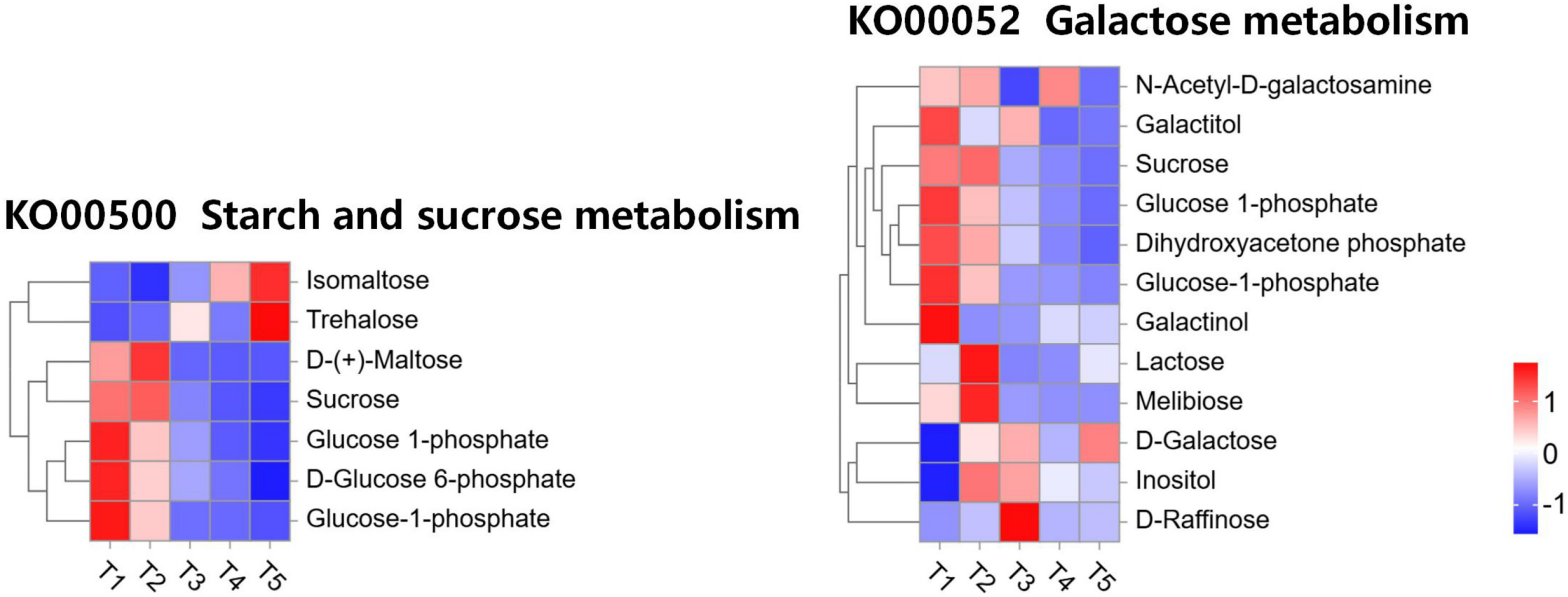
Accumulation patterns of DAMs involved in starch and sucrose metabolism and galactose metabolism. Heatmaps show DAMs mapped to ko00500 and ko00052. Red indicates higher accumulation, and blue indicates lower accumulation.

Based on KEGG annotation of the DEGs and carbohydrate metabolic pathways related to polysaccharide biosynthesis, ko00500 and ko00052 were further analyzed at the metabolite level. The results showed that both pathways were enriched with multiple metabolites closely associated with polysaccharide biosynthesis (Figure 9 and Supplementary Tables 3C,D). In general, most DAMs related to polysaccharide biosynthesis showed a gradually decreasing trend at later developmental stages. In ko00500, many sugars and phosphorylated intermediates directly linked to central carbon flux were relatively abundant at early stages. For example, maltose, sucrose, glucose-1-phosphate, and glucose-6-phosphate were upregulated at T1 or T2 and then gradually declined from T3 to T5. In contrast, trehalose and isomaltose were significantly lower during the early stages, but increased continuously with development and reached their highest levels at T5. This pattern suggests that, in the middle and late stages, carbon allocation may shift from readily utilizable transport sugars and metabolic intermediates toward disaccharide accumulation or carbon-buffering forms. In ko00052, metabolites showed an even clearer pattern of “early mobilization followed by mid/late depletion.” N-acetyl-D-galactosamine, galactitol, dihydroxyacetone phosphate, and glucose-1-phosphate were relatively high at T1 and significantly decreased thereafter, indicating stronger mobilization of galactose-related carbon sources and energy precursors during T1. Meanwhile, galactinol, lactose, dulcitol, and melibiose showed stage-specific increases at T2, suggesting that the young-fruiting-body stage may enhance the synthesis or accumulation of galactose-derived polyols and oligosaccharides to support cell-wall formation during rapid growth. Taken together, DAMs in both pathways indicate that *G. tsugae* enhances carbon-source mobilization and central carbon supply at early stages, whereas during and after basidiospore discharge most related sugar metabolites decline, accompanied by stage-specific enrichment of selected disaccharides or oligosaccharides, reflecting carbon redistribution and metabolic remodeling during late development.

#### 3.4.2 Joint mapping of DEGs and DAMs onto KEGG pathways

**FIGURE 10:**
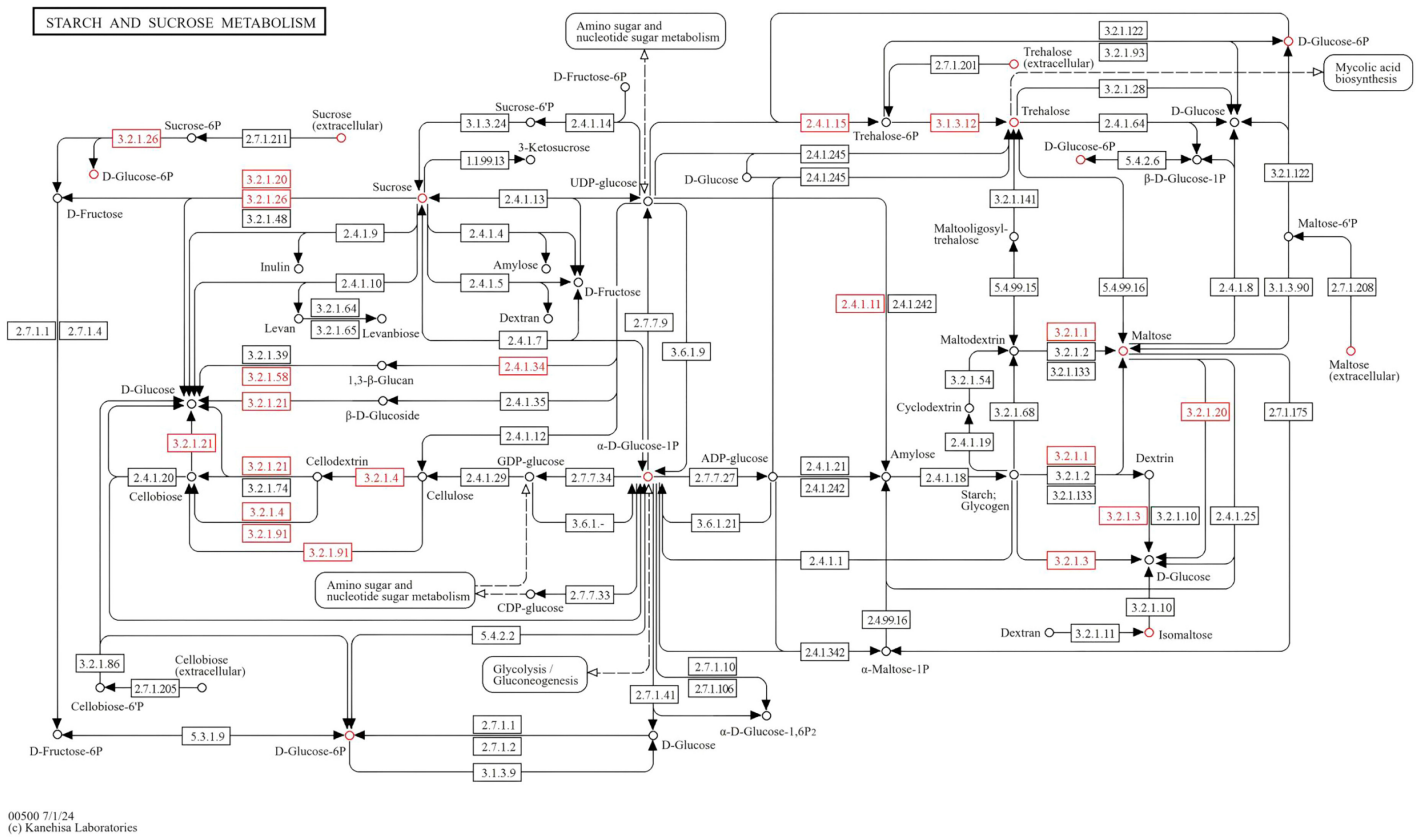
Mapping of DEGs and DAMs onto starch and sucrose metabolism pathway ko00500. Red boxes indicate DEG-annotated enzyme nodes, and red dots indicate mapped DAMs. Copyright permission obtained from Kanehisa Laboratories (Kanehisa et al., 2025). Source: KEGG PATHWAY database, ko00500, Starch and sucrose metabolism pathway.

KEGG annotation and enrichment analyses showed that DEGs were significantly enriched in ko00500 (Figure 10). This pathway is organized around sugar phosphates and nucleotide sugars such as UDP-glucose, α-D-glucose-1-phosphate (G1P), and glucose-6-phosphate (G6P), linking sucrose, starch, cellulose, and their degradation products (e.g., dextrins, maltose, and isomaltose) with central carbon pathways including glycolysis and gluconeogenesis. It therefore represents a key channel for polysaccharide degradation, monosaccharide supply, and precursor redistribution. Pathway mapping revealed that multiple reaction steps were associated with significantly differentially expressed enzyme-coding genes. Most of the annotated enzymes were glycoside hydrolases, including endo/exo-glucanases and β-glucosidases involved in cellulose and β-glucan degradation, as well as amylases and glucoamylases participating in the hydrolysis of starch, maltodextrins, and maltose. These results suggest that during development *G. tsugae* may enhance its ability to decompose structural and reserve polysaccharides, progressively converting complex polysaccharides into glucose and phosphorylated glucose forms that can enter central carbon metabolism directly.

Further mapping of DAMs onto ko00500 revealed strong agreement between the metabolomic and transcriptomic data (Figure 10). The enriched DAMs covered several key nodes, from disaccharides to monosaccharides and their phosphorylated intermediates, including sucrose, trehalose, maltose, isomaltose, D-glucose, glucose-1-phosphate, and glucose-6-phosphate. The dynamic fluctuations of these metabolites across developmental stages directly reflect stage-specific regulation of starch/cellulose degradation and sugar-phosphate redistribution. The pronounced accumulation of trehalose was especially notable, suggesting that during the middle and late stages this pathway may not only provide carbon sources but may also participate in carbon allocation strategies associated with fungal energy storage and stress adaptation. Overall, the coordinated changes in key enzyme genes and corresponding sugar metabolites in ko00500 indicate that *G. tsugae* undergoes pronounced carbohydrate mobilization and carbon-flux remodeling during development, thereby supplying carbon sources and precursors for cell-wall/extracellular polysaccharide biosynthesis and later developmental demands.

**FIGURE 11:**
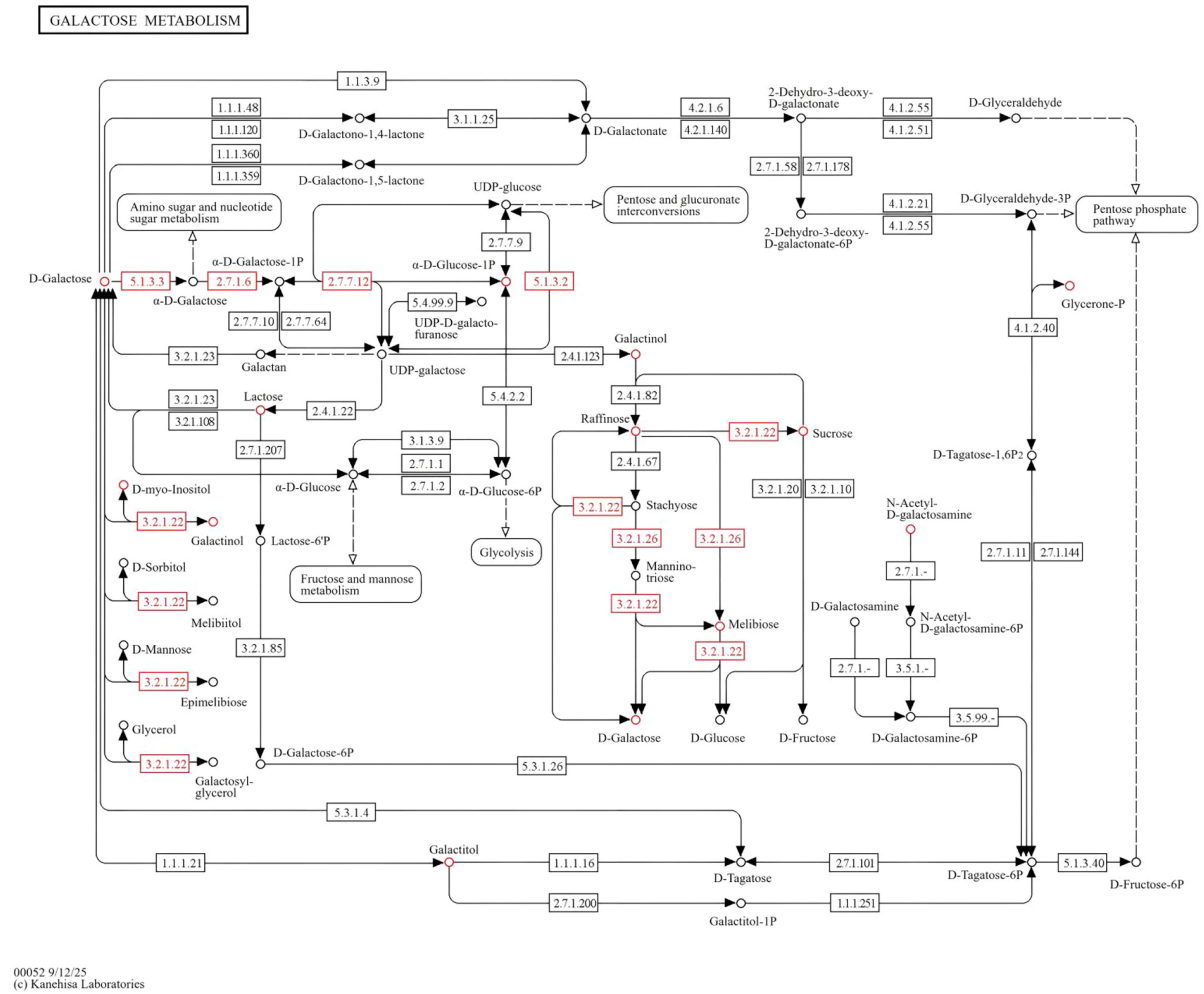
Mapping of DEGs and DAMs onto galactose metabolism pathway ko00052. Red boxes indicate DEG-annotated enzyme nodes, and red dots indicate mapped DAMs. Copyright permission obtained from Kanehisa Laboratories (Kanehisa et al., 2025). Source: KEGG PATHWAY database, ko00052, Galactose metabolism pathway.

Mapping DEGs to ko00052 showed that the mobilization and conversion of galactose-related carbon sources in *G. tsugae* were also subject to stage-specific regulation (Figure 11). In the upstream part of the pathway, lactose and raffinose-family oligosaccharides (e.g., raffinose, stachyose, and melibiose) can be progressively hydrolyzed by α-galactosidase and related glycoside hydrolases to release D-galactose, which then enters the classical Leloir pathway. D-Galactose is phosphorylated by galactokinase (GalK) to form galactose-1-phosphate (Gal-1P), which is subsequently converted by galactose-1-phosphate uridylyltransferase (GalT) into UDP-galactose and interconverted with UDP-glucose by UDP-glucose 4-epimerase (GalE). A key feature of this module is the efficient channeling of galactose released from external or structural oligosaccharides into the UDP-sugar nucleotide pool, thereby providing precursors for cell-wall and extracellular polysaccharide backbone elongation as well as galactose-containing side-chain donors. The pathway map revealed DEGs at several of these critical nodes, indicating coordinated transcriptional regulation of galactose-to-UDP-sugar conversion during development.

At the metabolite level, DAM mapping onto ko00052 identified multiple key nodes related to raffinose-family oligosaccharide mobilization and intermediate metabolism, including raffinose, stachyose, melibiose, and galactinol (Figure 11). These metabolites are located along the reaction chain that converts raffinose-family oligosaccharides into monosaccharides. Galactinol was also detected as a DAM and connected to raffinose-family nodes, indicating that DAMs covered multiple key branches of galactose-derived polyols and oligosaccharides. In addition to oligosaccharide nodes, DAMs were also detected at central monosaccharide and intermediate nodes, including D-galactose and its downstream branch points. Sucrose was likewise annotated on the pathway map and linked to the raffinose branch, indicating that DAMs were not confined to galactose-derived oligosaccharides but also involved connections with sucrose metabolism. Overall, the DAM mapping results for ko00052 showed that differential metabolites mainly covered key positions in the “galactosyl oligosaccharide-galactose-related sugar node” continuum.

### 3.5 qRT-PCR analysis

**FIGURE 12:**
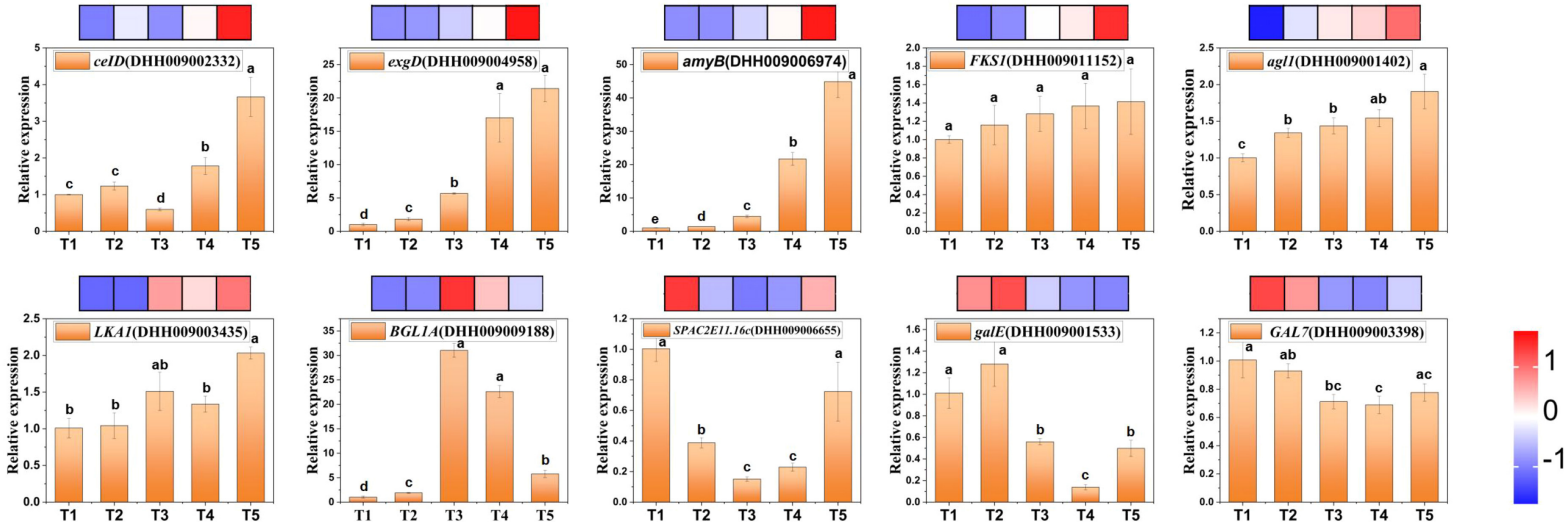
qRT-PCR validation of polysaccharide-related candidate genes in *G. tsugae*. Bar charts show relative expression levels determined by qRT-PCR, and heatmaps show corresponding RNA-seq expression patterns. 18S was used as the internal reference gene. Values are shown as mean ± SD. Statistical analysis was performed using ΔCt values, whereas relative expression levels were presented as 2^ΔΔCt^. Different lowercase letters indicate significant differences among developmental stages for each gene at *P* < 0.05. qRT-PCR, quantitative reverse transcription PCR.

Across the five developmental stages (T1–T5), 10 genes were selected for validation: *FKS1* and *exgD*, which directly participate in β-glucan chain synthesis/degradation; *celD* and *BGL1A*, which are involved in cellulose degradation; *LKA1* and *amyB*, which participate in starch degradation; *agl1*, which contributes to both starch and sucrose catabolism; *SPAC2E11.16c*, which is related to trehalose synthesis; and *galE* and *GAL7*, which participate in galactose conversion. The qRT-PCR results generally agreed with the expression trends observed in the RNA-seq data (Figure 12), confirming the reliability of the transcriptomic dataset. Gene-specific primers used for qRT-PCR are listed in Table 1.

**TABLE 1:** Gene-specific primers used for qRT-PCR validation.

KEGG pathway	Gene ID	Gene symbol	Forward primer	Reverse primer
ko00500	DHH009002332	*celD*	ACGGTCGGAAACTTGCTCTA	ATCTCCTGCATCGTAGTAGCC
ko00500	DHH009004958	*exgD*	CCACAGTTCCAATGTCGGC	TGTGGTAGGTGAAGATGCTCC
ko00500	DHH009006974	*amyB*	TCTTCGTCGCATCCGCTTTT	GCCTGTTGATGATACCCCTCC
ko00500	DHH009011152	*FKS1*	AGCGGCGTATCTCGTTCTTT	ACGAAGTTGTCCCACTCGAC
ko00500	DHH009001402	*agl1*	CACCACCGTGAACGAGACA	GATGAACGCCTTGACCTCCG
ko00500	DHH009003435	*LKA1*	TTATCACCGACCGCTACGC	CAAATCGCAGTGAAGCCTGC
ko00500	DHH009009188	*BGL1A*	TAAGCCGGGTACGACGAAAG	TCGTTGACGGGATCGTTTCT
ko00500	DHH009006655	*SPAC2E11.16c*	AGAATGCCATCGACTCCGTC	GTCGTAGCAGCTCGCAAAAT
ko00052	DHH009001533	*galE*	CCGAACTCAAACGGGTGCTA	GCGCGATCTTTGCGACG
ko00052	DHH009003398	*GAL7*	TTTTGACCCTACAACCCACCACCC	GACAGAGGTAGCACTTGGGGG

### 3.6 Correlation analysis between metabolome and transcriptome

**FIGURE 13:**
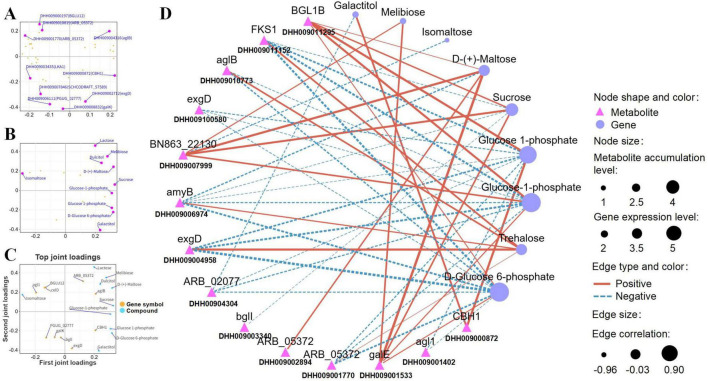
O2PLS loading plots and gene–metabolite correlation network. **(A)** Gene loadings. **(B)** Metabolite loadings. **(C)** Joint gene–metabolite loadings. **(D)** Spearman correlation network. Circles represent metabolites, triangles represent genes, red edges indicate positive correlations, and blue edges indicate negative correlations. O2PLS, two-way orthogonal partial least squares.

The preceding results showed that many DEGs and DAMs participated in pathways associated with polysaccharide biosynthesis, indicating that *G. tsugae* possesses a comprehensive and complex polysaccharide-biosynthetic activity network. To further evaluate the roles of these DEGs and DAMs, correlation analyses were performed. Gene loadings (Figure 13A) and metabolite loadings (Figure 13B) highlighted variables with the highest biological variation, whereas Figure 13C presents the top-ranked joint loadings. Genes and metabolites showed clear spatial clustering along the first and second joint loading dimensions, indicating strong coordination in their variation patterns. Specifically, in the positive region of the first joint axis (first joint loadings > 0), key candidate genes such as *aglB*, *CBH1*, *exgD*, and *ARB_05372* clustered tightly with sugar metabolites including sucrose, maltose, lactose, and glucose-1-phosphate. This spatial concordance suggests strong associations between carbohydrate-metabolism-related genes and the accumulation of these sugars. In contrast, in the negative region of the first loading, genes such as *aglI*, *BGLU12*, *celD*, and *bglL* showed a similar distribution pattern to isomaltose, indicating another metabolic branch mediated by specific glycosidases.

### 3.7 Integrated analysis of functional modules associated with polysaccharide biosynthesis

**FIGURE 14:**
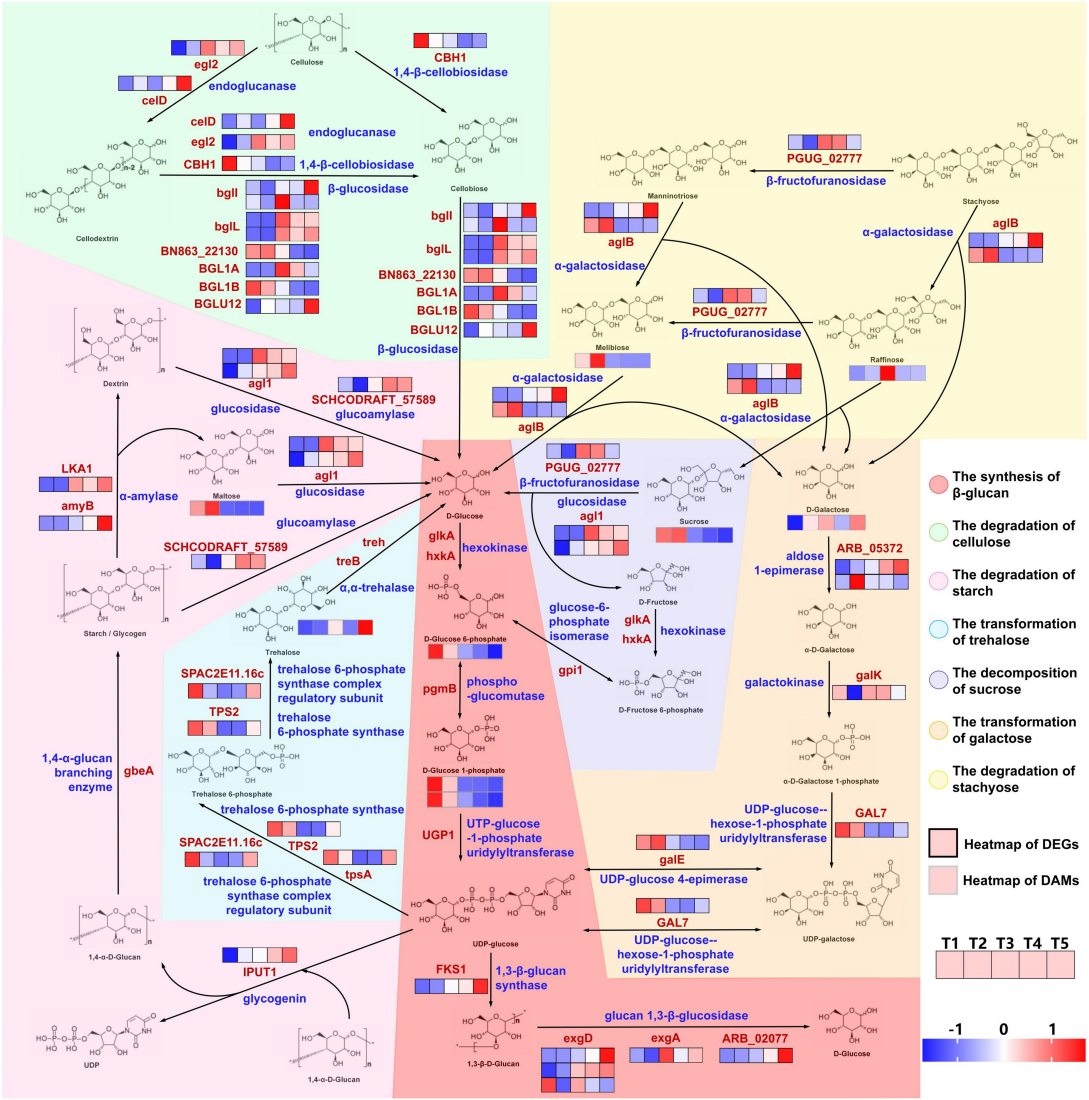
Manually reconstructed integrated pathway model showing dynamic changes in DEGs and DAMs involved in polysaccharide metabolism and biosynthesis in *G. tsugae*. The integrated pathway was manually reconstructed and edited using Microsoft PowerPoint based on KEGG pathway maps for starch and sucrose metabolism (ko00500) and galactose metabolism (ko00052). DEGs and DAMs identified in this study were mapped onto the corresponding pathway nodes. Colored regions indicate functional modules: β-glucan synthesis, cellulose degradation, starch/glycogen degradation, trehalose metabolism, sucrose decomposition, galactose transformation, and stachyose degradation. Small heatmaps show DEG expression or DAM accumulation patterns from T1 to T5. Red indicates higher expression or accumulation, and blue indicates lower expression or accumulation.

By integrating the two key pathways, the reaction routes corresponding to DEGs and DAMs were combined into a unified scheme, with corresponding gene symbols, enzymes, and metabolites annotated on the pathway map. Together with DEG and DAM heatmaps across the five developmental stages, this analysis yielded the functional pathway map shown in Figure 14. The results indicate that β-glucan biosynthesis in *G. tsugae* involves 35 DEGs, 8 DAMs, and 20 related enzymes. Among them, starch and sucrose metabolism (ko00500) includes five DAMs and 27 DEGs and involves 14 enzymes, whereas galactose metabolism (ko00052) includes five DAMs (two of which overlap with ko00500) and 10 DEGs (two of which also overlap with ko00500), involving six enzymes.

Overall, the polysaccharide metabolism and biosynthesis network of *G. tsugae* can be divided into seven functional regions. Within ko00500, five functional regions were distinguished: (1) the red region, representing the main β-glucan biosynthetic chain, in which glucose is converted into the important precursor UDP-glucose, subsequently used for glucan chain formation, after which the glucan can also be degraded back into glucose; (2) the green region, corresponding to cellulose degradation, in which cellulose is progressively decomposed into glucose; (3) the pink region, representing starch or glycogen degradation, in which exogenous starch or endogenous glycogen is broken down into glucose; (4) the blue region, representing trehalose metabolism, where trehalose can be synthesized from UDP-glucose and later degraded into glucose when required; and (5) the purple region, corresponding to sucrose degradation, in which sucrose is converted into glucose and fructose. Within ko00052, two functional regions were distinguished: (6) the orange region, representing galactose conversion, in which galactose is converted into UDP-galactose and linked to the main glucose-derived trunk through interconversion between UDP-galactose and UDP-glucose; and (7) the yellow region, corresponding to stachyose degradation, in which stachyose can be degraded into glucose and galactose, thereby feeding directly into the glucan biosynthetic trunk, entering β-glucan biosynthesis after galactose conversion, or yielding sucrose that can subsequently enter the glucan biosynthetic process through sucrose degradation.

In the red region (1), six DEGs corresponded to two enzymes, namely the β-1,3-glucan synthase encoded by *FKS1* and the exo-1,3-β-glucosidases encoded by *ARB_02007*, three *exgD* genes, and *exgA*. In addition, four related genes did not meet the differential-expression threshold but all showed TPM values above 50, suggesting sustained high expression throughout fruiting body development. Based on their functional positions, β-1,3-glucan synthase and exo-1,3-β-glucosidase are likely to be important candidate enzymes in this region. This region mainly comprises three successive reaction steps: (i) sugar activation, in which glucose is phosphorylated by hexokinase using ATP to form glucose-6-phosphate and is then converted by phosphoglucomutase into glucose-1-phosphate; (ii) precursor generation, in which glucose-1-phosphate is converted into UDP-glucose by UDP-glucose pyrophosphorylase with consumption of UTP; and (iii) chain elongation, in which UDP-glucose serves as a key precursor for progressive β-glucan assembly by β-glucan synthase. Thus, the red region should represent one of the key reaction modules in *G. tsugae* polysaccharide biosynthesis.

In the green region (2), 14 DEGs were involved in cellulose degradation, indicating transcriptional responses related to cellulose utilization at different developmental stages. Overall, these genes did not exhibit identical expression trends, suggesting that *G. tsugae* may deploy distinct cellulose-degradation strategies at different stages. Notably, *CBH1*, which catalyzes cellulose degradation to cellobiose, showed higher expression at earlier stages and lower expression at later stages, whereas egl2 and celD, which degrade cellulose to cellodextrins, showed the opposite pattern, with low early expression and higher late expression. This result suggests that the dominant enzyme types responsible for cellulose degradation may differ between developmental phases.

In the pink region (3), the four DEGs involved in starch degradation all showed relatively low expression at early stages, indicating that starch-related degradation may be less active during early development of *G. tsugae*. Overall, their expression levels increased progressively as development proceeded. Meanwhile, IPUT1 encodes a glycogenin-like protein that may participate in the incorporation of UDP-glucose into the 1,4-α-glucan chain, followed by the action of the 1,4-α-glucan branching enzyme encoded by *gbeA* in glycogen synthesis, and glycogen may subsequently be degraded to release glucose. These observations suggest that starch- and glycogen-related metabolism may jointly contribute to carbon-source regulation during later development.

In the blue region (4), three DEGs were involved in trehalose synthesis, whereas two non-DEGs participated in trehalose degradation to glucose, indicating that genes associated with trehalose metabolism are present throughout all stages. The three trehalose-synthesis genes were highly expressed at early stages and decreased markedly during middle development, indicating dynamic stage-dependent changes in trehalose-related metabolism. The accumulation of trehalose was relatively low in the early stage and significantly increased in the later stage, which might indicate that trehalose was more decomposed into glucose for energy supply and the formation of the fruiting body morphology in the early stage.

In the purple region (5), three DEGs were associated with sucrose degradation and were expressed at higher levels during the middle and late stages, suggesting that sucrose catabolism may be more active during these periods. Sucrose degradation yields glucose and fructose, of which glucose can enter the main polysaccharide biosynthetic trunk directly, whereas fructose must first be phosphorylated and converted into glucose-6-phosphate before entering the trunk pathway. Most sucrose may originate from the substrate, but some may also derive from stachyose degradation in the yellow region (7). Therefore, sucrose may be an important intermediate metabolite linking starch and sucrose metabolism (ko00500) with galactose metabolism (ko00052).

The orange region (6) and yellow region (7) belong to galactose metabolism (ko00052). Three DEGs were related to stachyose degradation, two of which were *agl1* genes encoding α-galactosidase. Because galactose is released during stachyose degradation, changes in the yellow region (7) may affect further galactose conversion in the orange region (6). The conversion of galactose to UDP-glucose is mainly mediated by galK, GAL7, and galE. These three genes showed relatively high expression at early stages, suggesting that *G. tsugae* may enhance the conversion of galactose to UDP-sugar precursors early in development, thereby providing supplementary carbon sources for β-1,3-glucan biosynthesis.

## 4 Discussion

This study showed that crude polysaccharide content in *G. tsugae* fruiting bodies followed a characteristic developmental pattern, increasing gradually at early stages, peaking at mid-development, and then declining slightly at later stages. The highest content occurred at T3, whereas T1 and T5 showed relatively low levels. Combined with previous reports on *G. lucidum* (Gao et al., 2024; Ma et al., 2025) and other basidiomycetes (Nagy et al., 2023), this trend likely reflects shifts in carbon-allocation strategies across development. During T1, carbon resources are mainly invested in mycelial growth and substrate decomposition, and polysaccharide biosynthesis remains preparatory. During T2–T3, carbon flux is increasingly directed toward cell-wall and extracellular polysaccharide synthesis, leading to marked polysaccharide accumulation. During T4–T5, polysaccharides may be partially degraded to satisfy the energetic demands of spore formation and tissue maintenance, while biosynthetic capacity simultaneously declines as the fruiting body enters senescence; together, these changes likely explain the observed decrease in polysaccharide content. It should be noted that this study did not purify or structurally identify polysaccharides with specific structural definitions such as β-D-glucans or glycoprotein components containing pyrophosphate reported in previous studies. Therefore, the polysaccharides discussed here should be understood as crude polysaccharide components or biological synthesis characteristics related to polysaccharides, rather than purified and structurally identified active polysaccharides.

Mechanistically, starch and sucrose metabolism (ko00500) constitutes the core carbon-flux route underlying polysaccharide accumulation in *G. tsugae*. Differential expression of *FKS1* and multiple glycoside hydrolase-related genes, together with dynamic changes in glucose-1-phosphate, glucose-6-phosphate, sucrose, and maltose, indicates that polysaccharide biosynthesis in *G. tsugae* is not a single polymerization reaction but rather a coordinated system coupling precursor supply, glycan-chain elongation, and cell-wall polysaccharide remodeling. Recent functional studies have further confirmed this perspective. In *G. lucidum*, both the β-1,3-glucosyltransferase gl20535 (Liu et al., 2025) and the α-1,3-glucosyltransferase glagt (Chen et al., 2024) can remodel polysaccharide yield and composition by affecting UDP-glucose supply, expression of glycosyltransferases or glycoside hydrolases, and cell-wall status. Therefore, the T3 peak in polysaccharide content is more plausibly explained not by the isolated enhancement of a single biosynthetic enzyme, but by an optimal coordinated state in which precursor supply is abundant, relevant enzymes are active, and polysaccharide formation proceeds efficiently.

Another key finding was that the utilization of exogenous substrate and the mobilization of endogenous carbon reserves appear to be partitioned across developmental stages. *CBH1* was highly expressed early, whereas cellulose-degradation-related genes such as celD were enhanced later. Together with the observation that rapidly utilizable sugars in ko00500 were abundant early but decreased later, this suggests that *G. tsugae* relies more strongly on substrate decomposition to obtain utilizable carbon sources during primordia formation and early fruiting body growth, and gradually shifts toward structural remodeling and remobilization of endogenous reserves during basidiospore discharge and post-ripening. A study of continuous growth cycles in *G. lucidum* likewise found that the expression and activity of lignocellulose-degrading enzymes changed significantly with development and were closely associated with the accumulation of carbohydrates and active constituents in fruiting bodies (Zhou et al., 2018). At the same time, studies in other basidiomycetes have shown that β-1,3-glucanase participates in β-glucan degradation in late developmental or postharvest stages, suggesting that the mild decline in polysaccharide content at late stages more likely reflects intensified remodeling and consumption rather than a complete loss of biosynthetic ability (Konno et al., 2014). This interpretation provides a clearer biological explanation for the observed decrease in polysaccharide content during T4 to T5: carbon flux in late development shifts from accumulation toward release and reproduction.

Results related to trehalose further support this carbon-redistribution model. Trehalose and isomaltose accumulated progressively during the middle and late stages, whereas glucose-1-phosphate and glucose-6-phosphate declined, indicating that carbon is not simply depleted later in development but is increasingly redirected into sugar forms associated with buffering and maintenance of homeostasis. Transcriptomic analysis of basidiospore discharge in *G. lucidum* has shown that genes related to trehalose synthesis are significantly upregulated during mature spore formation, suggesting that trehalose is closely associated with spore development and late-stage carbon storage (Cai et al., 2021). More direct functional evidence has shown that *GlGSK3* can phosphorylate and activate trehalose-6-phosphate synthase, thereby improving trehalose production, ATP metabolic efficiency, and thermotolerance (Wang et al., 2025). Accordingly, the increased trehalose level during late development of *G. tsugae* probably reflects the establishment of energy homeostasis, cellular protection, and a rapidly mobilizable carbon pool during basidiospore discharge and post-ripening, rather than passive enhancement of a single biosynthetic branch.

In contrast, galactose metabolism (ko00052) appears to function more as a supplementary carbon-supply and structural-diversity module than as the dominant trunk pathway. The relatively high expression of galK, GAL7, and galE at early stages suggests that, when carbon flux through the main glucose pathway is constrained, galactose-related branches may provide additional UDP-sugar precursors. Genomic analysis of *G. tsugae* has shown that this species possesses the genetic basis for adapting to coniferous substrates and has strong substrate-degradation potential (Jiang et al., 2021). Meanwhile, galactose-rich bioactive polysaccharides have been isolated from *G. lucidum*, indicating that galactose may not only serve as a supplementary precursor carbon source but also contribute to structural diversification of the final polysaccharide products (Wu et al., 2025). Therefore, although the signal intensity of ko00052 was weaker than that of ko00500, its significance should not be underestimated. It suggests that polysaccharide accumulation in *G. tsugae* is controlled not only by developmental stage but also by the type of carbohydrate substrate available. This finding provides a theoretical basis for future use of agricultural by-products rich in raffinose-family sugars (e.g., soybean meal and wheat bran) as cultivation substrates to selectively activate this pathway and improve polysaccharide production.

## 5 Conclusion

The present study demonstrates that crude polysaccharide accumulation in *G. tsugae* fruiting bodies shows pronounced stage specificity, reaching its highest level at pre-basidiospore discharge and then declining slightly during basidiospore discharge and post-ripening. Integrated multi-omics analysis further indicates that this pattern is closely associated with transcriptional reprogramming and metabolic remodeling. Starch and sucrose metabolism (ko00500) constitutes the core carbon-flux pathway for polysaccharide biosynthesis, whereas galactose metabolism (ko00052) functions as a supplementary module for carbon supply and structural diversification. Coordinated changes in FKS1 and multiple glycoside hydrolase-related genes, together with sugar phosphates and disaccharides, suggest that polysaccharide accumulation in *G. tsugae* is a coupled process involving substrate utilization, precursor generation, glycan-chain elongation, and late-stage remodeling. From an application perspective, these findings identify T3 as a promising developmental window for stage-directed harvesting, quality evaluation, and subsequent structure-activity-guided utilization of *G. tsugae* polysaccharides. However, because this study focused on crude polysaccharide content and multi-omics associations, the specific structures and bioactivities of T3-derived polysaccharides remain to be determined. Future studies integrating polysaccharide purification, monosaccharide composition analysis, molecular-weight determination, glycosidic-linkage analysis, FT-IR/NMR characterization, and antioxidant or immunomodulatory assays will be necessary to validate the functional significance of polysaccharide fractions accumulated at T3. Overall, these findings provide an important foundation for understanding the developmental regulation of polysaccharide formation in *G. tsugae* and for future precision cultivation, substrate optimization, and functional validation of candidate genes.

## Data Availability

The transcriptome sequencing data presented in this study have been deposited in the NCBI Sequence Read Archive (SRA) repository under BioProject accession number PRJNA1470220. The raw sequencing reads are available under SRA Run accession numbers SRR38820000–SRR38820014.

## Footnotes

1
https://github.com/OpenGene/fastp

2
http://bowtie-bio.sourceforge.net/bowtie2/index.shtml

3
http://daehwankimlab.github.io/hisat2/

4
https://ccb.jhu.edu/software/stringtie/

5
https://deweylab.github.io/RSEM/

6
https://bioconductor.org/packages/DESeq2/

7
http://classyfire.wishartlab.com/

8
https://www.r-project.org/

9
http://classyfire.wishartlab.com/

10
https://www.kegg.jp/
