# Supplementary material for: Multi-omics analysis of polysaccharide accumulation and associated metabolic reprogramming across developmental stages of Ganoderma tsugae
Source: Front Microbiol. 2026 Jun 17;17:1856773. doi: 10.3389/fmicb.2026.1856773 (PMC13319048; doi:10.3389/fmicb.2026.1856773)
Supplement: Supplementary file 4 [file Data_Sheet_1.docx]

*
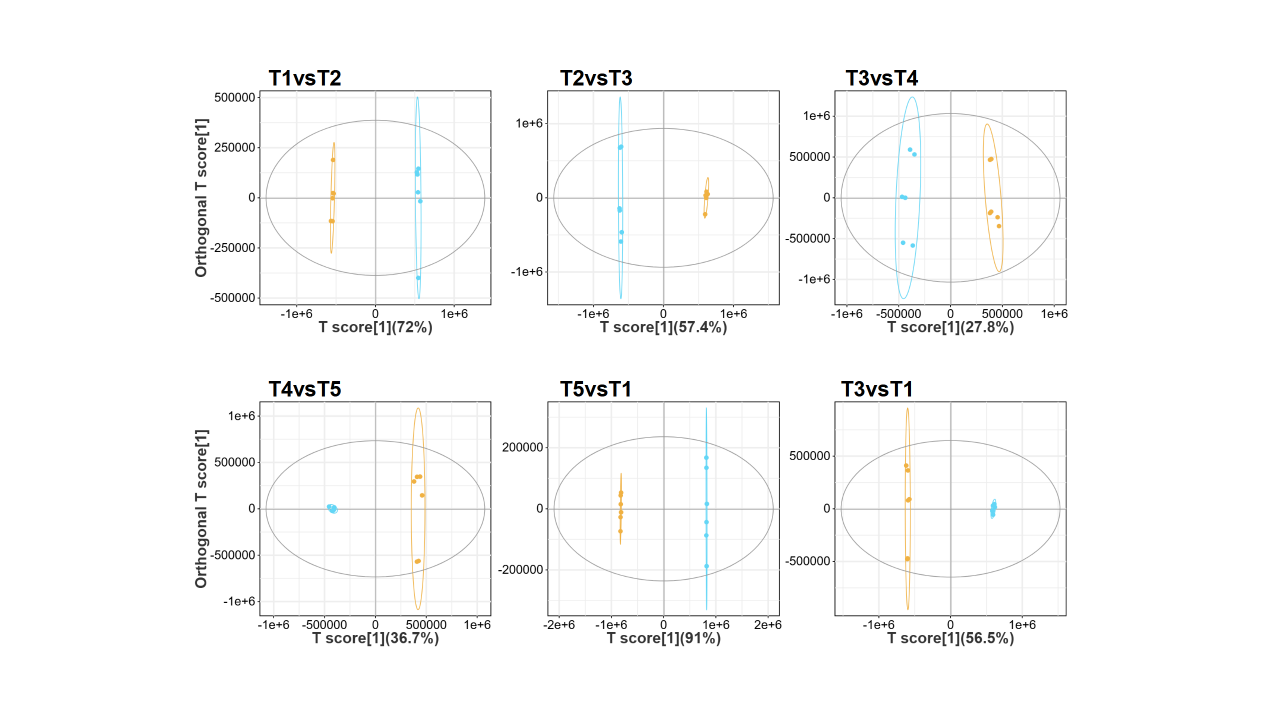
*

*Supplementary Fig. S1. OPLS-DA score plots of metabolites from G. tsugae across developmental stages.*

*
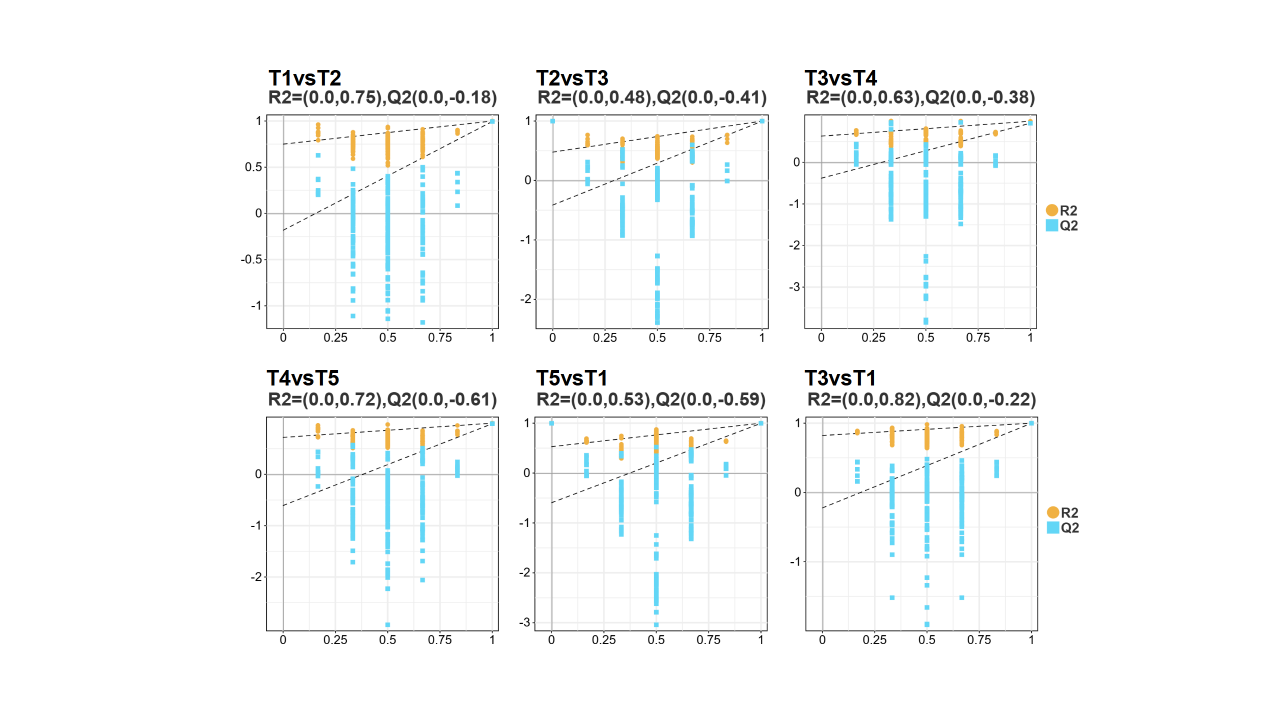
*

*Supplementary Fig. S2. Permutation tests for metabolite OPLS-DA models across developmental stages of G. tsugae.*
